# Unveiling the Promise: Navigating Clinical Trials 1978–2024 for PDAC

**DOI:** 10.3390/cancers16213564

**Published:** 2024-10-23

**Authors:** Angel A. Dominguez, Matthew T. Perz, Yi Xu, Leonor G. Cedillo, Orry D. Huang, Caitlin A. McIntyre, Vignesh Vudatha, Jose G. Trevino, Jun Liu, Pei Wang

**Affiliations:** 1 Department of Cell Systems & Anatomy; University of Texas Health Science Center at San Antonio, San Antonio, TX 78229, USA; domingueza6@livemail.uthscsa.edu (A.A.D.); perzm@livemail.uthscsa.edu (M.T.P.); xuy4@uthscsa.edu (Y.X.); leocedill@gmail.com (L.G.C.); orry.huang@utexas.edu (O.D.H.); liuj8@uthscsa.edu (J.L.); 2 Division of Surgical Oncology and Endocrine Surgery, University of Texas Health Science Center at San Antonio, San Antonio, TX 78229, USA; mcintyrec1@uthscsa.edu; 3 Department of Surgery, Virginia Commonwealth University School of Medicine, Richmond, VA 23298, USA; vignesh.vudatha@vcuhealth.org (V.V.); jose.trevino@vcuhealth.org (J.G.T.)

**Keywords:** PDAC, clinical trials, treatment

## Abstract

Despite many decades of research, pancreatic ductal adenocarcinoma (PDAC) remains one of the most difficult cancers to diagnose and treat effectively. Although there have been improvements in the 5-year overall survival rate, it is still very low at 12.5%. The limited efficacy of current therapies, even when PDAC is detected early, underscores the aggressive nature of the disease and the urgent need for more effective treatments. Clinical management of PDAC still relies heavily on a limited repertoire of therapeutic interventions, highlighting a significant gap between research efforts and available treatments. Over 4300 clinical trials have been or are currently investigating different treatment modalities and diagnostic strategies for PDAC, including targeted therapies, immunotherapies, and precision medicine approaches. These trials aim to develop more effective treatments and improve early detection methods through advanced imaging techniques and blood-based biomarkers. This review seeks to categorize and analyze PDAC-related clinical trials across various dimensions to understand why so few chemotherapeutic options are available to patients despite the numerous trials being conducted. This review aims to provide a comprehensive and nuanced understanding of the landscape of PDAC-related clinical trials, with the overarching goal of identifying opportunities to accelerate progress in drug development and improve patient outcomes in the fight against this devastating disease.

## Simple Summary

Pancreatic ductal adenocarcinoma (PDAC) is one of the most challenging cancers to diagnose and treat. Despite over 4,300 clinical trials for pancreatic cancer exploring various treatments, progress in developing effective options for PDAC remains slow. This review seeks to offer a detailed and insightful analysis of the current landscape of PDAC-related clinical trials, with the primary goal of identifying ways to expedite drug development and enhance patient outcomes in the battle against this aggressive disease.

## 1. Introduction

Pancreatic ductal adenocarcinoma (PDAC) is the most prevalent type of pancreatic cancer, accounting for approximately 90% of all cases. PDAC has long been one of the most challenging cancers to diagnose and treat effectively. Despite many decades of intensive research, significant improvements in the 5-year survival rate have only recently emerged, climbing from a dismal 2% to a slightly more hopeful 12.5% [1]. However, nearly half of PDAC cases are diagnosed after the cancer has already metastasized, leading to a 3% 5-year survival rate for this cohort. Even when detected in its early stages, PDAC remains difficult to treat, with a 5-year survival rate of only 44% [1]. This striking statistic underscores the aggressive nature of PDAC and the limited efficacy of current therapeutic approaches. Compounding these challenges is the fact that PDAC is often diagnosed late in its progression, when treatment options are limited, and the disease has already established a foothold.

Given the urgent need to improve outcomes for PDAC patients, research efforts have intensified in recent years, focusing on the development of novel therapies and diagnostic tools [2,3]. Despite this push, progress has been slow. There are two primary cytotoxic chemotherapy regimens used in the United States, gemcitabine plus nab-paclitaxel and FOLFIRINOX, [4] and while these treatments can extend survival in certain patients, they are often associated with significant side effects and limited efficacy. There are additional agents that can be used in specific subsets of patients with identified actionable mutations, such as PARP inhibitors in patients with BRCA1/2 mutations or in KRAS wildtype patients [5,6].

**Figure 1:**
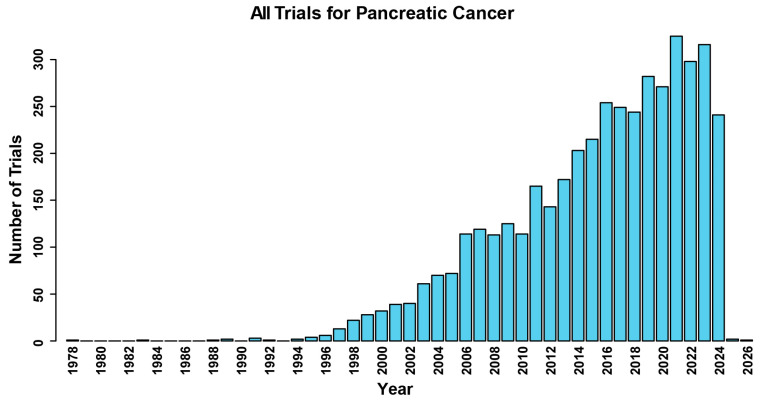
The number of trials related to pancreatic cancer each year since 1978.

To address the shortcomings with current therapeutic approaches and late-stage diagnosis, researchers have embarked on over 4300 clinical trials aimed at exploring new treatment modalities and diagnostic strategies for pancreatic cancer (Figure 1). These trials encompass a wide range of approaches, including targeted therapies, immunotherapies, and precision medicine approaches tailored to individual patients’ genetic profiles. Additionally, efforts are underway to improve early detection methods for PDAC, including the development of novel imaging techniques and blood-based biomarkers. Despite the challenges, the growing understanding of PDAC biology and the rapid pace of technological innovation offer hope for future breakthroughs in the diagnosis and treatment of this devastating disease. Continued investment in research and collaboration among scientists, clinicians, and patients will be essential to obtain the goal of improving outcomes for PDAC patients in the years to come.

The increasing landscape of clinical trials dedicated to PDAC reflects a concerted effort within the scientific and medical communities to address the pressing need for more effective treatment options for this aggressive malignancy. However, despite the growing volume of research activity, the clinical management of PDAC remains largely reliant on a limited repertoire of therapeutic interventions. Understanding the discrepancy between the number of clinical trials and the few major advances in pancreatic cancer treatment is crucial for guiding future research endeavors as well as designing better clinical trials. In pursuit of this objective, this review seeks to undertake a comprehensive categorization and analysis of PDAC-related clinical trials across various dimensions.

A comprehensive review of active and recent clinical trials for PDAC (as found in ClinicalTrials.gov) can provide several valuable insights into the field. Firstly, it allows researchers to track the progress of ongoing and completed trials, providing insights into the latest advancements and emerging trends in PDAC treatment. Secondly, it helps identify gaps in current research, guiding future studies to address unmet needs and explore novel therapeutic approaches. Thirdly, understanding the diversity of intervention types, including drug trials, immunotherapies, and combination treatments, can inform the development of more effective treatment strategies. Ultimately, this comprehensive analysis supports the continuous improvement of PDAC therapies, aiming to enhance patient survival and quality of life.

## 2. Brief Review on PDAC

### 2.1. PDAC Staging

Pancreatic cancer is typically staged with the American Joint Committee on Cancer (AJCC) TNM system [7,8]. This staging system is crucial for determining the best treatment approach and providing a prognosis. The “T” in TNM stands for the tumor’s size and extent of invasion into nearby tissues. The “N” indicates whether and how many nearby lymph nodes are affected by the cancer. The “M” represents metastasis, indicating whether the cancer has spread to other parts of the body. The TNM values are then correlated with the AJCC stages, which range from 0 to 4. Stage 0, also known as carcinoma in situ (sometimes referred to as Tis), includes precancerous conditions where the tumor has not begun to invade other tissues. This stage is critical for potential intervention and treatment before the cancer progresses. Stage 4 signifies advanced cancer with at least one distant metastasis, indicating that the cancer has spread beyond the primary site to other organs, which significantly complicates treatment options and worsens the prognosis.

The tumor stage at diagnosis is directly related to overall prognosis. Evaluation of the Surveillance, Epidemiology, and End Results (SEER) database [9,10] provides valuable data on pancreatic cancer, tracking survival rates based on the extent of disease spread. While the overall survival rate is 12.8%, patients with localized cancer (Stage I and IIA) have a 5-year survival rate of 44%, those with regional disease in the lymph nodes (Stage IIB-III) have a 5-year overall survival rate of 16% (stage III), and those with distant metastases (Stage IV) have an overall survival rate of only 3%. The marked decline in survival rates as cancer progresses underscores the urgency for advances in early detection techniques. Early detection could drastically improve survival rates by enabling interventions at a stage when the cancer is more localized and treatment options are more effective. Research into early diagnostic tools and treatment strategies remains a priority in the fight against pancreatic cancer, aiming to shift the diagnosis toward earlier stages and improve overall outcomes.

### 2.2. PDAC Genetics

Genetics play a significant role in the development and progression of PDAC, influencing both hereditary and sporadic forms of the disease [11]. Over the last decade, the genomic landscape has been better elucidated [12], and understanding the genetic landscape of PDAC has led to the discovery of actionable mutations and subsequent use of targeted therapies [13,14,15,16]. There are four commonly altered genes in PDAC, *KRAS*, *TP53*, *CDKN2A,* and *SMAD4*, with many other genes altered at lower frequencies [13,17]. The most altered gene in PDAC is *KRAS*, which is mutated in over 90% of cases [18]. The most frequent mutation of *KRAS* is at codon 12, where glycine is substituted by another amino acid such as aspartate, valine, or cysteine [19]. This leads to constitutive activation of KRAS, and subsequent downstream signaling resulting in uncontrolled cell proliferation. This uncontrolled cell growth is a key driver of cancer development and progression. Mutations in the tumor suppressor gene *TP53* are found in approximately 50–75% of PDAC cases [20], which contributes to the evasion of cell death and genomic instability. *CDKN2A* gene is another tumor suppressor and is inactivated in 30–50% of cases [21]. Mutations or deletions in *SMAD4*, which occur in about 55% of PDAC cases, are associated with advanced disease and metastasis [22].

Additionally, there are several key pathways which are altered in PDAC [16], which are beyond the scope of this review. For example, mutations homologous recombination pathway (i.e., BRCA1, BRCA2, PALB2) have been increasingly understood, and may respond better to platinum-based chemotherapies and PARP inhibitors [5,6].

### 2.3. Diagnosis of PDAC

Pancreatic cancer is challenging to diagnose and often remains undetected until later stages when the prognosis is far poorer, making diagnostic tools critical in reducing mortality [23]. Imaging techniques currently employed to detect PDAC include multidetector computed tomography (MDCT), positron emission tomography (PET), magnetic resonance imaging (MRI), and endoscopic ultrasound (EUS) appearance [1]. Imaging is typically performed when there is a suspicion of pancreatic cancer based on a patient’s symptoms, rather than as a screening tool. Diagnosis is established with a biopsy, with an endoscopic approach more commonly used.

Biomarkers for early detection of pancreatic cancer are limited. Tumor markers are commonly obtained when a diagnosis of pancreatic cancer is suspected, most commonly CA19-9 [24] and CEA [25]. CA19-9, or carbohydrate antigen 19-9, is a tumor marker often used in the context of gastrointestinal cancers, particularly pancreatic cancer. CA 19-9 is not used as a primary diagnostic tool due to its lack of specificity and sensitivity, but it can support the diagnosis of pancreatic cancer in conjunction with other diagnostic methods such as imaging and/or biopsy [26]. Many studies have evaluated novel tissue and circulating biomarkers, including exosomes [27], miRNA [28], glycoproteins [29], MIC-1 [30], and ctDNA as well as many others [31], yet their use in the clinical setting is limited and they remain exploratory.

Screening for pancreatic cancer is not routinely performed, and it is challenging to detect it [32]. However, in patients who are thought to be at high risk for developing pancreatic cancer, such as those with a known germline mutation, screening is recommended. If screening is recommended, it is most common to use a combination of EUS and MRI for high-risk patients.

### 2.4. Treatment

The primary systemic treatment options for PDAC consists of cytotoxic chemotherapy agents. Gemcitabine became the standard of care for PDAC shortly after FDA approval in 1997 [33]. It is a cytidine analog that can be incorporated into DNA or RNA as gemcitabine triphosphate. Gemcitabine diphosphate also serves to inhibit ribonucleotide reductase [34]. However, gemcitabine is rendered less effective because tumor-associated macrophages and fibroblasts release deoxycytidine, which competes with gemcitabine at the enzyme that would otherwise incorporate it into DNA. Moreover, some forms of PDAC have demonstrated the ability to detoxify gemcitabine directly with cytidine deaminase [2]. A 2019 study found that patients with pancreatic cancer treated with gemcitabine had a mean overall survival of 2 years and a 5-year survival rate of 15–20% [35].

Nab-Paclitaxel was first approved as a treatment in combination with gemcitabine for late-stage pancreatic cancer in 2013 [36]. The “nab” component, albumin, allows the drug to accumulate in tumor areas by binding SPARC (secreted protein, acidic and rich in cysteine) and travel more easily through cells [37]. Paclitaxel itself stabilizes the assembly state of microtubules during cell division, arresting them in the G2 or M phase, and eventually causing apoptosis [38]. Patients treated with nab-Paclitaxel in combination with gemcitabine were found to have a longer median overall survival (8.5 months) and higher survival rate (35%) when compared to patients treated with just gemcitabine (6.7 months and 22%, respectively) [36].

5-Fluorouracil (5-FU) was first approved by the FDA in 1962 for the treatment of colorectal cancer [39]. It was compared to gemcitabine in a 1997 study; although gemcitabine would become the first-line treatment due to better tolerance, 5-FU continued to be employed both as a monotherapy and in combination therapies [40,41]. 5-FU is an antimetabolite analog of uracil that disrupts RNA synthesis and inhibits thymidylate synthase. The inhibition of thymidylate synthase leads to an accumulation of uracil and a reduction in available thymine, resulting in the incorporation of uracil or 5-FU metabolites into DNA. Efforts to repair this damaged DNA further harm the cells, causing strand breaks and eventual cell death.

FOLFIRINOX, a combination of folinic acid (FOL) (also referred to as leucovorin or calcium folinate), fluorouracil (F), irinotecan (IRIN), and oxaliplatin (OX), was established as a first-line treatment for pancreatic cancer in 2011, when it was found to have superior survival compared to gemcitabine monotherapy [42]. Folinic acid enhances the effectiveness of 5-FU by stabilizing the binding of 5-FU to the enzyme thymidylate synthase. Irinotecan is a topoisomerase inhibitor that prevents DNA from unwinding and duplicating, leading to DNA damage and cell death. Oxaliplatin is a platinum-based drug that forms cross-links in DNA, preventing DNA replication and transcription, ultimately leading to cell death. However, concerns about FOLFIRINOX’s toxicity have driven further exploration into dose optimization and investigations into how the regimen can be further improved through changes to its pharmacokinetics [43,44]. For instance, a recent study found that NALIRIFOX, a combination of folinic acid, fluorouracil, liposomal irinotecan, and oxaliplatin, demonstrated an efficacy comparable to FOLFIRINOX with a different profile of adverse effects [45], possibly due to liposomal irinotecan’s increased permeability and retention at the tumor site compared to non-liposomal irinotecan [46].

## 3. All Trials for Pancreatic Cancer

The Food and Drug Administration Modernization Act of 1997 requires any NIH-funded clinical trial to register with ClinicalTrials.gov [47]. According to this database, the first registered clinical trial concerning pancreatic neoplasms took place in 1978 [NCT00001165], when researchers investigated the impact of the combination chemotherapy 5-fluorouracil and doxorubicin in patients with Zollinger-Ellison Syndrome and metastatic non-beta-islet cell neoplasm. Since then, the number of clinical trials for potential therapeutic interventions for pancreatic cancer has markedly increased. As of June 2024, 4398 clinical trials related to pancreatic cancer have been registered with ClinicalTrials.gov (Figure 1) (Supplemental Table S1). Despite the rise in research activity in recent years, significant advances in treatment that have been seen in other cancers have not been seen for patients who are diagnosed with pancreatic cancer.

**Figure 2:**
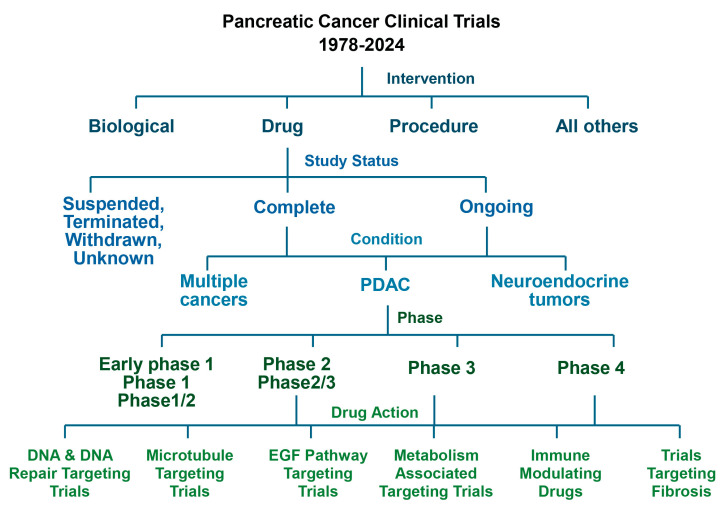
Organization of the analysis of pancreatic cancer clinical trials.

In this review, we aim to evaluate the trials conducted in the field of PDAC. We exported all clinical trials related to “pancreatic cancer” without filtering out any information. We organized the trials based on the filter categories defined by ClinicalTrials.gov. We first stratified them based on “Intervention” and further focused on “Drug” along with their “study status”. We then specifically analyzed PDAC only for both “Complete” and “Ongoing” trials. Only Phase 2 and higher trials were further analyzed based on drug action (Figure 2).

### 3.1. Interventions

**Figure 3:**
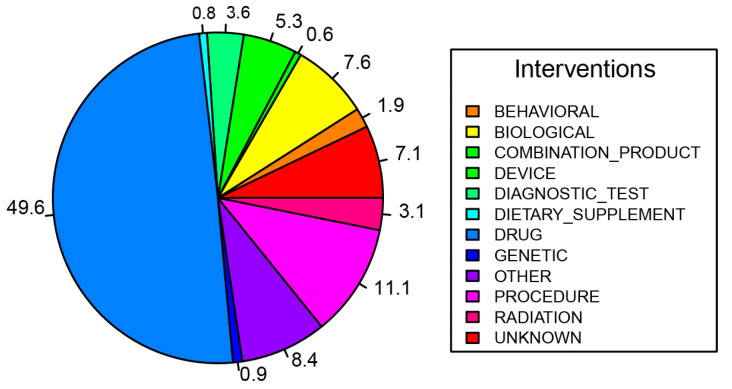
Various Interventions in clinical trials of pancreatic cancer.

A myriad of interventions have been explored to better diagnose and treat PDAC. These interventions have been grouped based on their respective methodologies and mechanisms of action (*n* = 4398) (Figure 3). Within these various intervention methods, which may overlap with others, we have defined the major areas of study within each group to illustrate which aspects of PDAC and its treatment have been studied the most intensely.

### 3.2. Behavioral

Behavioral studies constitute approximately 2% of all clinical trials for PDAC (n = 84). The majority of these studies occur in post-operative settings and focus on stress levels, coping behavior, engagement with others, anxiety, sleep, depression, drug susceptibility, and overall quality of life for both patients and caregivers. These studies primarily aim to understand patient responses to diagnosis, surgery, or treatments, and how interventions can facilitate or mitigate adverse events. They also explore how lifestyle impacts therapeutic outcomes and how certain treatments affect the patient’s lifestyle. Monitored criteria include muscle mass and strength, daily life changes (e.g., smoking abstinence), common chemotherapy side effects (e.g., vomiting and nausea), and mood assessments.

### 3.3. Biological

Biological interventions, representing approximately 8% of all PDAC clinical trials (n = 335), mostly include adoptive cell transfer (ACT) procedures, novel antibody administrations, and cancer vaccines. ACT procedures typically utilize chimeric antigen receptor (CAR) T cells, engineered to target specific antigens independent of major histocompatibility complex (MHC) engagement with T cell receptors (TCR). Other cells used in ACT treatments include CAR-NK cells, pulsed dendritic cells, and activated proinflammatory macrophages. Antibody-based treatments, the largest portion of biological interventions, antagonize protein interactions (e.g., anti-PD-1/L1, anti-CTLA-4), serve as agonists (e.g., 4-1BB), or target cancer cells for antibody-dependent cellular cytotoxicity (ADCC). Novel targets for PDAC include CD73 and TGF-β, which affect the immunosuppressive tumor microenvironment (TME). Lastly, cancer vaccines, comprising the smallest portion of biological interventions for PDAC, strengthen the patient’s immune system to combat cancer in an antigen-specific manner. This process often involves priming immune cells with the help of antigen-presenting cells (APCs) such as dendritic cells, macrophages, or B cells, to target cancer cells expressing these antigens.

### 3.4. Combination Product

Combination product interventions are defined as utilizing multiple methods to target cancer cells. Many clinical trials often utilize multiple drug intervention agents in combination with one another; however, this section primarily focuses on the use of non-therapeutic mechanisms combined with therapeutic intervention to monitor patient response. Studies that focus on these combinations (n = 27, <1%) will combine biological, pharmaceutical, radioactive, and dietary interventions with select procedures. A number of these studies primarily study stereotactic body radiation in combination with various therapeutic interventions.

### 3.5. Device

Devices may be used to aid in diagnosis, treatment, or disease monitoring. This section includes 231 clinical trials, many of which focus on novel methods to obtain biopsies or deliver drugs, with some devices having interventional characteristics themselves. Novel devices used for direct intervention might be utilized in ablation-based therapies such as cryoablation or radiant heat ablation; others involve irreversible electroporation. Some devices have been designed to implant intratumoral radioactive material preoperatively, while others are involved in obtaining samples in a minimally invasive manner.

### 3.6. Diagnostic Test

Studies on the development of diagnostic testing (n = 157) largely focus on optimizing current strategies or developing novel methods. As PDAC is usually diagnosed at a late stage due to late clinical manifestations, early detection methods present an opportunity to significantly impact treatment success. Some studies focus on discovering novel biomarkers for early disease detection from serum (e.g., ctDNA, cfDNA). Others enhance PET/CT/MRI scans with drugs to improve detection. Combining novel imaging strategies with biomarkers could promote early detection, predict treatment response, and improve overall survival.

### 3.7. Dietary Supplement

Less than 1% (n = 34) of PDAC clinical trials focus solely on the impact of dietary supplements on disease progression and therapeutic efficacy. Some studies investigate nutritional plans and their potential influence on perioperative procedures. Several studies examine the impact of surgical or therapeutic interventions on patient nutrition, while others focus on immune cell abundance, proinflammatory cytokines, and overall survival. These studies may guide clinicians in developing patients’ nutritional plans that promote postoperative recovery, improve quality of life, and mitigate nutritional or caloric deficiency.

### 3.8. Genetic

Genetic interventions (n = 38; approximately 1%) mainly focus on identifying mutations associated with PDAC to better understand the biology and progression of the disease. They test the efficacy of targeted therapies aimed at specific genetic alterations and explore the role of genetic biomarkers in predicting treatment response and patient outcomes. Additionally, these trials investigate the potential of personalized medicine approaches, such as tailoring treatments based on a patient’s genetic profile and examining the genetic epidemiology of PDAC, to uncover hereditary risk factors.

### 3.9. Procedural

Procedural interventions make up approximately 11% of all PDAC clinical trials (n = 486). These trials evaluate new surgical techniques and their efficacy in tumor removal, assessing histopathological responses to different procedures and monitoring postoperative recovery. They also study the objective response and quality of life post-surgery, and the tumor uptake of imaging-facilitating molecules. Additionally, these trials investigate adverse events associated with surgical interventions and the integration of procedural techniques with other therapies like chemotherapy or radiation. The goal is to optimize surgical outcomes and enhance overall treatment efficacy.

### 3.10. Radiation

About 138 clinical trials involving radiation therapy for PDAC focus on several key aspects. They assess the efficacy of radiation in shrinking tumors and improving survival rates, often in combination with chemotherapy (chemoradiation) or novel therapies like immunotherapy. These trials also explore optimal radiation doses and advanced techniques such as Stereotactic Body Radiation Therapy (SBRT) to maximize tumor control while minimizing side effects. Additionally, they investigate the role of radiation in palliative care to alleviate symptoms and improve quality of life in advanced PDAC. Monitoring and managing side effects is another crucial aspect of these trials.

### 3.11. Other

Over 8% (n = 371) of PDAC clinical trials categorized under “other” for PDAC often explore diverse and innovative approaches that do not fit neatly into conventional categories. These trials investigate the use of advanced imaging technologies for better diagnosis and monitoring, explore novel drug delivery systems, and assess the impact of lifestyle modifications such as diet and exercise on disease progression and patient outcomes. Additionally, they evaluate the role of supportive care interventions, such as pain management techniques, psychological support, and integrative medicine practices, to improve the overall quality of life for patients.

### 3.12. Drugs

Drug interventions represent the largest portion of interventional trials, encompassing almost half of all interventions (n = 2183; 49.6%). These drug interventions include a wide variety of methods and targets such as novel chemotherapies, targeted therapies, imaging agents’ safety, immunotherapies, repurposed drugs, and small molecule inhibitors. Most studies use standard drug treatments in combination with novel options. The current standard treatments (gemcitabine, 5FU, and nab-paclitaxel) serve as comparison points for new treatments. These interventions typically examine the maximum tolerated dose, overall survival, objective response, and synergistic effects. The primary measures vary by trial phase and will be explored in the following section. In addition, since trial registrations on ClinicalTrials.gov are conducted by various entities, including different pharmaceutical companies and research groups, information on the drug action and target is often not included in the registration. This omission makes it difficult to navigate and identify specific targets. To aid in this process, we compiled a list of drugs used in the analyzed trials (Supplemental Table S2) for easier reference.

## 4. Drug Intervention Analysis

Clinical trials aim to test the efficacy, potency, and other medical interventions or observations of particular agents or methodologies in humans. These trials are conducted in various phases: early phase 1, phase 1, phase 1/II, phase 2, phase 2/3, phase 3, and phase 4. Early phase 1 involves limited human exposure to collect initial safety and pharmacokinetic data. Phase 1 assesses safety, dosage, and side effects in small cohorts (15–30 patients). Phase 1/II trials involve smaller cohorts (<100 patients) to focus on safety, dosage, and preliminary efficacy. Phase 2 evaluates efficacy and further assesses safety in 100–300 patients. Phase 2/3 confirms efficacy and compares it with current treatments. Phase 3 compares experimental treatments to current standard treatments in large groups (1000–3000 patients) to gather evidence on efficacy, efficiency, and side effects for FDA approval. Phase 4 tests licensed drugs in large groups over long periods to monitor long-term side effects and efficacy.

As drug interventions are the most abundant in all PDAC clinical trials, this group will be analyzed in greater detail to fully understand drug interventions throughout various phases. Focusing on drug intervention analysis not only aids in the discovery and development of new treatment options, but also enhances our understanding of the biological mechanisms underlying PDAC. This comprehensive analysis can guide future research directions, optimize clinical trial designs, and ultimately contribute to the development of more effective and personalized therapeutic strategies for PDAC patients.

### 4.1. Drug Clinical Trial Study Status

Based on the study status, we categorized them into three groups: “Suspended, Terminated, Withdrawn and Unknown” group, “Complete” group and “Ongoing” group. We will then discuss the trials by different phases.

### 4.2. Suspended, Terminated, Withdrawn, and Unknown Drug Trials

A quarter of drug trials (n = 585) have the study status of “Suspended”, “Terminated”, “Withdrawn”, or “Unknown” (STWU). Trials may be terminated, withdrawn, or suspended due to reasons such as toxicity, severe adverse events, harmful effects, or lack of clinical efficacy. Many interventions fail within phase 1 trials due to toxicity. Trials that move forward to phase 2 are evaluated for clinical efficacy but can fail due to previously unknown toxicity, adverse effects, or lack of efficacy. Interventions that fail during phase 3 often do so due to insufficient evidence supporting clinical efficacy.

**Figure 4:**
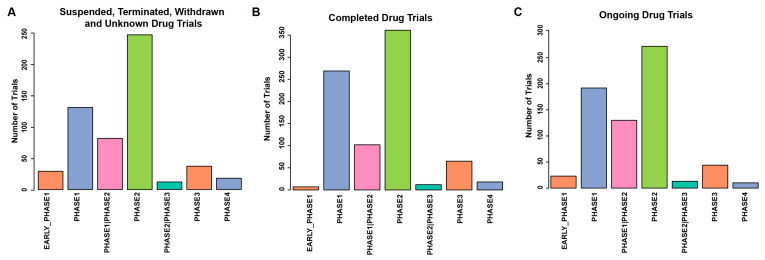
Number of different phase trials in STWU group (**A**), completed group (**B**) and ongoing group (**C**).

Phase 1 trials, including early phase 1, account for 159 STWU trials, with only 13 posting study results (8.2%). Phase 2 contains the greatest number of failed trials, with 244 failures in PDAC treatments, 77 of which posted study results (31%). Only 37 interventions progressing to phase 3 have failed, with 8 posting study results (21%). Eighteen phase 4 trials have failed, with 2 results posted (11%) (Figure 4A). Overall, only 21% of all trials had study results posted after SWTU. It is unfortunate when clinical trials end in this group. Posting the study results on how these trials failed may provide valuable information to guide future trial designs.

### 4.3. Completed Drug Trials

Clinical trials with the study status “completed” indicate that the trial has finished its intended research activities, including participant enrollment, intervention administration, and follow-up. These trials have gathered all necessary data and are in the process of analyzing results to draw conclusions. Compared with STWU trials, a larger fraction, but not all, of the completed trials eventually posted their trial results. The completion status provides a clearer picture of the intervention’s safety, efficacy, and overall impact, contributing valuable information to the medical community and potentially influencing future clinical practices and treatment guidelines.

As trials progress, they must complete each phase before advancing, or not advancing, to the next phase. A total of 876 trials have been completed, with only 269 (31%) posting results. Of the 277 early phase 1 or phase 1 trials, 32 (12%) posted results. Phase 2 trials (including phase 2 and phase 1/II) have the highest completion rate with 463 trials, 204 of which (44.1%) had study results posted. Phase 3 saw 65 completions with 23 (35%) posted study results, and phase 4 had 18 completions with 8 (44.4%) posted results (Figure 4B). Overall, only 31% of all trials were marked “yes” for study results after completion, with phase 2 being the most common for completion and phase 4 having the highest percentage of posted results.

### 4.4. Ongoing Drug Trials

As of June 2024, amazingly, 1342 pancreatic cancer clinical drug trials are ongoing trials, as “Active not recruiting” (n = 168), “Recruiting” (n = 971), and “Not yet recruiting” (n = 202). “Active not recruiting” describes a trial that has recruited participants and is ongoing. “Recruiting” indicates that the trial is currently seeking participants. “Not yet recruiting” refers to trials that have not started recruiting participants. Currently, there are 278 ongoing clinical trials at early Phase 1 and Phase 1. Phase 1/2 and Phase 2 contain 469 ongoing trials. Phase 2/3 and Phase 3 have 70 ongoing clinical trials. Phase 4 has 13 ongoing clinical trials (Figure 4C). Detailed analysis of these trials will be in a later section.

## 5. Analysis of Completed Drug Trials

We chose to focus the analysis on a portion of the completed trials. We excluded Phase 1 trials and investigated Phase 2 to Phase 4 trials (N = 456). Many of these clinical trials test drugs in multiple cancers, so we focused our analysis on the 355 trials specifically for PDAC. One of filters describes the diseases involved in the clinical trial as “Condition” which has used different names for PDAC such as “Advanced Pancreatic Cancer”, “Pancreatic Neoplasm” etc. Various names for PDAC make it difficult to categorize them. The 23 trials that include the commonly used medications (i.e., antibiotics, pain relievers, and vitamins) with the primary aim to improve patient quality of life, and the additional 124 trials, which evaluated dosages, combinations, or various formulations of current cytotoxic chemotherapy agents, were not discussed here. We focus on the 208 trials that investigate unique drugs or combinations of novel drugs with standard chemotherapies including gemcitabine (G), gemcitabine/nab-paclitaxel (P), and FOLFIRINOX (F) (Supplemental Table S3).

### 5.1. DNA and DNA Repair Targeting Trials

**Figure 5:**
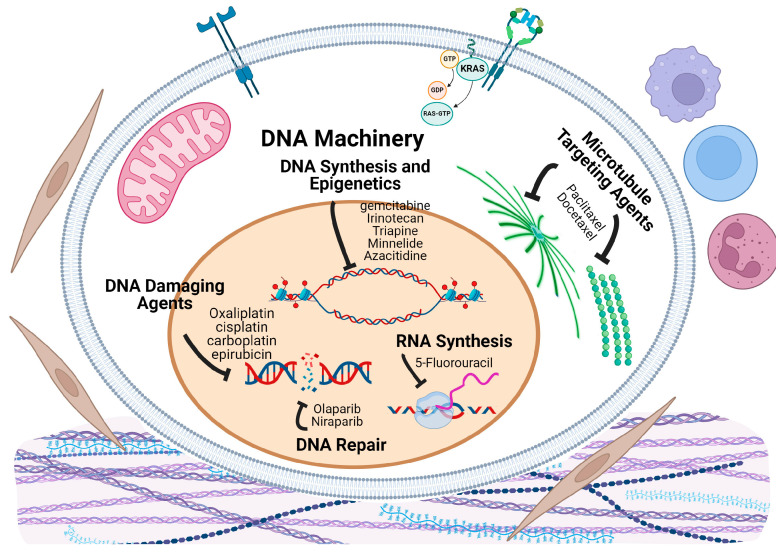
Cellular processes inhibited by DNA and microtubule targeting agents.

Cancer cells have higher rates of DNA replication and cell division compared to normal cells. Thus, a common strategy to target cancer cells is to induce DNA damage or disrupt DNA synthesis or repair machinery (Figure 5), and PDAC is no exception. Alkylating agents, such as cisplatin [48], which add alkyl groups to DNA and lead to DNA damage and cell death, are still being tested in many phase 2 and 3 clinical trials. DNA repair targeting agents are largely focused on PARP inhibitors. Niraparib, Olaparib, and Rucaparib have completed phase 2 trials, with Olaparib being the only PARP inhibitor to complete phase 3. Inducing overwhelming DNA damage in cancer cells results in an exit from the cell cycle and entry into apoptosis; however, there is evidence that genotoxic drug treatments may cause cells to enter mitosis [49]. These types of drugs are still in their infancy for treating PDAC due to the difficulty of infiltrating the tumor microenvironment to target tumor cells directly.

### 5.2. DNA-Damaging Agents

DNA damaging agents include alkylating and intercalating agents such as cisplatin, carboplatin and epirubicin. Cisplatin, which forms covalent bonds with DNA and creates crosslinks, was evaluated in 11 separate trials, of which only three have published results. In a phase 2 trial [NCT00490360] [50] involving 28 patients with resectable pancreatic cancer, patients received 1000 mg/m^2^ of gemcitabine and 50 mg/m^2^ of cisplatin in four bi-weekly cycles. Tumor response was significant, with 54% of patients showing a response and 83% displaying cytopathic effects, resulting in a median overall survival (OS) of 26.5 months. Another phase 2 study [NCT00335543] [51] examined the temporal influence of cisplatin on surgical resection of pancreatic cancer. In this study, 254 patients were divided into 2 arms. Arm A patients underwent primary surgery before chemotherapy, while Arm B patients received neoadjuvant chemotherapy followed by surgical resection. The results showed a 4.33-month increase in median OS, with Arm A having a median OS of 18.9 months and Arm B having a median OS of 25.0 months.

Other DNA damaging drugs, epirubicin and carboplatin, were also evaluated in phase 2 trials. In one study [NCT01150630] [52], 98 patients were divided into three arms. Arms A and B received 30 mg/m^2^ of cisplatin and 1250 mg/m^2^ of epirubicin, with arm A undergoing treatment for 14 days every two weeks for 6 months, and arm 2 receiving the same treatment for 3 months. Arm C received 1000 mg/m^2^ for three weeks every four weeks. Although the published data did not include median OS, the treatment demonstrated moderate efficacy, measured by the percentage of patients’ event-free at one year: 23% for Group A, 50% for Group B, and 66% for Group C. Researchers concluded that the neoadjuvant addition was safe and tolerable, warranting further phase 3 trials for these drugs.

In summary, while cisplatin and other DNA-damaging drugs can be effective for certain PDAC patients, particularly those with specific genetic profiles, their overall benefit is limited by resistance and toxicity. Continued research is needed to optimize their use and identify patients who are most likely to benefit from these treatments.

### 5.3. DNA Synthesis and Epigenetic Inhibitors

Drugs targeting enzymes involved in DNA synthesis have completed phase 2. Ribonucleotide reductase (RNR) is an enzyme responsible for catalyzing the formation of deoxyribonucleotides, the precursors for DNA synthesis. Three trials have targeted this enzyme [53,54,55]. Triapine has demonstrated high efficiency in inhibiting RNR and was used in all three completed phase 2 trials. Additionally, a greater understanding of epigenetics has allowed for potential therapeutic targeting of epigenetic proteins. Two trials have targeted epigenetic mechanisms at phase 2. Minnelide, a small molecule anti-superenhancer that functions to inhibit MYC completed phase 2 in 2017 [56]. Azacitidine is a drug that inhibits the methylation of DNA to change gene expression and completed its phase 2 trial [NCT01845805] [57] in early 2021. This trial had 49 randomized participants: 24 received 300 mg of Azacitidine daily for 28 days along with first-line chemotherapy, while the remaining 25 participants received first-line chemotherapy alone as an observation group. The overall survival (OS) for the observation group was determined to be 26.4 months, whereas the treatment group had an OS of 33.8 months. Although there appears to be an increase in OS, there was no significant increase in OS or time to relapse. To gain further insight into the clinical efficacy of this drug, epigenetically profiling patients may be beneficial.

### 5.4. DNA Repair Inhibitors

The only completed phase 3 trial investigating the use of an agent that targets the DNA repair pathway was the POLO trial. This trial compared maintenance therapy with a PARP inhibitor, Olaparib, versus a placebo in a double-blind randomized controlled trial in patients with metastatic pancreatic cancer who had received first-line platinum-based chemotherapy and a germline BRCA1/2 mutation [NCT02184195] [58,59]. This trial included 154 participants; the intervention group (n = 92) received 300 mg of Olaparib twice daily as compared to the control group (n = 62). Initial results of the trial demonstrated that median progression-free survival (PFS) was longer in patients who received Olaparib as compared to those who received placebo (7.4 months versus 3.8 months; HR 0.53; 95% CI 0.35 to 0.82, *p* = 0.004). A follow up analysis demonstrated no significant difference in median overall survival between groups (19 months in Olaparib group versus 19.2 months in the placebo group; HR: 0.83; 95% CI: 0.56 to 1.22; *p* = 0.3487). Severe adverse events in the Olaparib group were seen 31.1% (n = 28), as compared to 16.4% (n = 10) in the placebo group). This study did not demonstrate clinical efficacy in increasing overall survival; however, the hazard ratio (HR) favored Olaparib, indicating clinical significance in terms of time away from standard treatments.

### 5.5. Microtubule Targeting Trials

Microtubules are one of the main components of the cytoskeleton, involved in a multitude of cellular processes including cellular division, cellular morphology, intracellular transport and motility [60]. They are composed of tubulin heterodimers that dynamically grow and shrink as required for cellular activities. During cell division, particularly in anaphase, microtubules rapidly polymerize and depolymerize to pull chromatids to opposite poles of the cell. Microtubule-targeting agents (Figure 5) can stabilize or destabilize microtubules, hindering cell division and promoting apoptosis in cancer cells. Targeting microtubule dynamics has proven to be an effective form of anti-proliferative therapy in cases of metastatic breast cancer, lung cancer, prostate cancer, and ovarian cancer [61,62,63,64].

Paclitaxel binds to the β-tubulin subunit of microtubules, promoting their assembly and inhibiting their disassembly [65]. This stabilization disrupts the dynamic reorganization of microtubules necessary for mitosis, effectively halting cell division at the metaphase-anaphase transition and triggering apoptotic cell death due to prolonged mitotic arrest. This mechanism has proven effective in treating PDAC in its nanoparticle albumin-bound formulation (nab-paclitaxel). Nab-paclitaxel, often combined with gemcitabine, enhances therapeutic effects in PDAC by overcoming the dense stromal environment of pancreatic tumors, improving drug delivery and penetration [45]. It has become one of the standard chemotherapies for PDAC.

More drugs targeting microtubules have been developed and there have been 18 completed phase 2 and 1 phase 3 trials testing them in PDAC. Docetaxel has been involved in various phase I and II trials, in combination with gemcitabine and capecitabine. The phase 2 trial [NCT01459614] [27] explored the efficacy and safety of a four-drug regiment GTX-C (capecitabine, gemcitabine, docetaxel, and cisplatin) in newly diagnosed untreated metastatic pancreatic cancer patients. The results showed a 6-month progression-free survival rate of 74.2% and an overall disease control rate of 89%. Median progression-free survival was 8.4 months, and overall survival was 12.9 months. The study concluded that GTX-C is highly active and well-tolerated, warranting further investigation in larger comparative studies.

### 5.6. Epidermal Growth Factor and Downstream Pathway Targeting Trials

**Figure 6:**
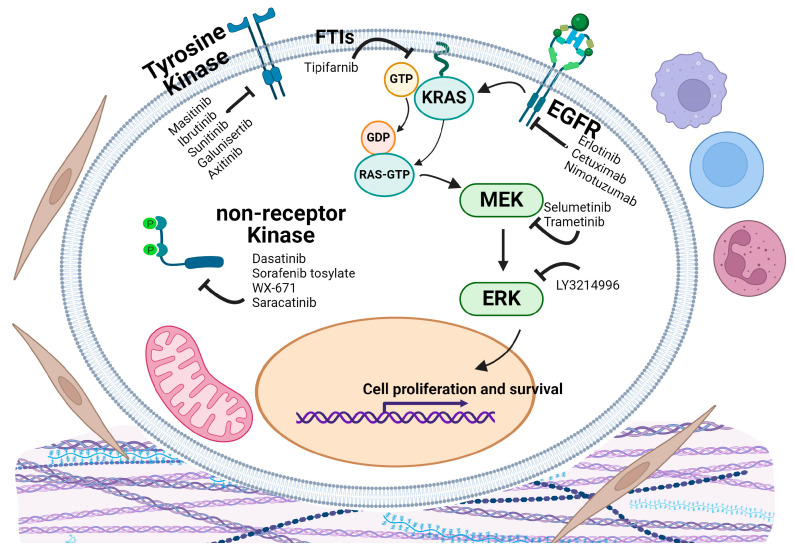
EGFR and tyrosine kinase inhibitors. (Created with BioRender.com).

The Epidermal Growth Factor Receptor (EGFR) pathway is crucial in regulating cell growth, survival, proliferation, and differentiation, and is often dysregulated in PDAC [66] (Figure 6). Overexpression or abnormal activation of EGFR in PDAC promotes tumor growth, angiogenesis, invasion, and metastasis. Although mutations in EGFR are less common in PDAC, its amplification and overexpression are frequently observed, leading to enhanced signaling through downstream pathways such as KRAS [67]. Cancers that are dependent upon the EGFR pathways have shown great response to EGFR inhibition combined with chemotherapy, such as colorectal cancers, cancer of the head and neck, and HER2+ breast cancer [68]. Thus, twenty-five phase 2 and four phase 3 clinical trials specifically tested EGFR inhibitors either alone or combined with standard chemotherapy for PDAC. Erlotinib has been used in more than half of these trials [69]. Antibody drugs Cetuximab [70] and Nimotuzumab [71] have also been tested.

### 5.7. EGFR Inhibitors

Erlotinib is a small molecular inhibitor targeting EGFR tyrosine kinase [72]. A phase 3 double blind study [NCT00026338] [73] aimed to evaluate the anti-PDAC efficacy of erlotinib in combination with gemcitabine. In this trial, 569 participants with unresectable or metastatic disease were randomly assigned to two arms in a 1:1 ratio. The first arm received standard gemcitabine treatment along with 100–150 mg/daily of erlotinib administered orally. The second arm received gemcitabine with a placebo. The combination treatment group had a median OS of 6.24 months, compared to 5.91 months in the gemcitabine-alone group. Notably, the one-year survival rate was 23% in the erlotinib group versus 17% in the placebo group. Additionally, there was a significant increase in progression-free survival in those who received erlotinib (*p* = 0.004) compared to the placebo group. This trial demonstrated erlotinib can significantly improve survival in patients with advanced pancreatic cancer. Given the results of the previous clinical trial, erlotinib was further investigated in the phase 2 RACHEL study [NCT00652366] [74] involving 467 patients to assess its potential for improved clinical efficacy against pancreatic cancer. The study concluded that increasing the dose of erlotinib did not enhance its efficacy.

The use of anti-EGFR antibody cetuximab, in addition to a standard care regimen including chemoradiation and chemotherapies, was evaluated in a phase 2 trial [NCT00338039] [75]. In this trial, 69 participants underwent treatment with gemcitabine (1 g/m^2^), oxaliplatin (100 mg/m^2^), cetuximab (500 mg/m^2^), and capecitabine (825 mg/m^2^) along with radiotherapy (50.4 Gy/28 fractions). Only 4 out of the 69 participants (5.8%) experienced severe adverse reactions, and a median overall survival (OS) of 19.2 months was observed. One year post-treatment, 66% of participants were alive; at two years, 25% were alive; and at four years, 11.3% of participants were still alive. This trial, among others, demonstrated that EGFR inhibitors are a critical and beneficial addition to the standard treatment of PDAC.

Out of four phase 3 trials, only two have publications associated with the trial number. In the phase 3 trial [NCT02395016] [76] involving 82 eligible patients with K-Ras wild-type tumors, participants were randomly assigned to receive either Nimotuzumab (400 mg once per week) or a placebo, followed by gemcitabine (1000 mg/m^2^ on days 1, 8, and 15, once every 4 weeks) until disease progression or unacceptable toxicity. The results showed that the median OS was 10.9 months for the nimotuzumab group compared to 8.5 months for the placebo group. Median progression-free survival (PFS) was 4.2 months for the nimotuzumab group versus 3.6 months for the placebo group (*p* = 0.04). Both OS and PFS were longer in the nimotuzumab group. The objective response rates were 7% for nimotuzumab versus 10% for placebo, and disease control rates were 68% versus 63%, respectively. The conclusion is that nimotuzumab plus gemcitabine significantly improved OS and PFS with a good safety profile in patients with locally advanced or metastatic KRAS wild-type PDAC.

The other phase 3 trial [NCT00040183] [77] has identified HER2 as a potential predictive biomarker for response to erlotinib treatment. Patients with higher HER2 levels had improved survival when treated with erlotinib (median OS 8.2 vs. 5.0 months), whereas patients with lower HER2 levels showed no significant difference in survival (median OS 6.0 vs. 8.3 months). This suggests that selecting patients based on HER2 levels could enhance the efficacy of erlotinib in PDAC treatment.

### 5.8. ERK and MEK Inhibitors

The ERK (Extracellular signal-Regulated Kinase) and MEK (MAPK/ERK Kinase) pathway are downstream of the EGFR and are frequently dysregulated in PDAC [78]. This pathway is often activated in PDAC due to mutations in upstream components, particularly KRAS, which has been considered non-druggable. Thus, ERK inhibitors such as LY3214996 [79], as well as MEK inhibitors like trametinib and selumetinib [80], have been tested in clinical trials. However, challenges such as drug resistance and toxicity remain significant hurdles. Multiple clinical trials are ongoing to determine optimal dosing, combination strategies, and identify biomarkers for patient selection. While some trials have shown promising results, the overall impact on long-term survival is still being evaluated, making this a crucial area of ongoing research in PDAC treatment.

In the phase 2 trial for MEK inhibitor [NCT00372944] [81], a total of 70 patients received either 100 mg oral selumetinib twice daily or 1250 mg/m^2^ oral capecitabine twice daily for 2 weeks followed by a 1-week break, in 3-week cycles. The median survival was 5.4 months in the selumetinib group and 5.0 months in the capecitabine group (*p* = 0.92). Disease progression occurred in 84% of patients in the selumetinib group and 5.0 months in the capecitabine group (*p* = 0.92). Disease progression occurred in 84% of patients in the selumetinib group and 88% in the capecitabine group. Even though selumetinib was well tolerated with a manageable safety profile, there was no statistically significant difference in overall survival between selumetinib and capecitabine as second-line treatments for advanced pancreatic cancer.

Another phase 2 trial [NCT01231581] [82] evaluated the efficacy of MEK inhibitor trametinib (GSK1120212), in combination with gemcitabine. 160 patients received either intravenous gemcitabine (1000 mg/m^2^) plus trametinib or gemcitabine plus a placebo. The study found no significant difference in OS between the treatment arms (HR 0.98; 95% CI, 0.67–1.44; *p* = 0.453), with median OS being 8.4 months for the trametinib group and 6.7 months for the placebo group. The study concluded that adding trametinib to gemcitabine did not improve OS and PFS in patients with metastatic pancreatic cancer.

### 5.9. KRAS Inhibition

The post-translational modification that adds a farnesyl group to the cysteine residue at the C-terminus of RAS proteins is a crucial step for the proper localization of RAS proteins to the cell membrane [83]. Farnesyltransferase inhibitors (FTIs) were developed as a potential therapeutic strategy to inhibit KRAS function [84,85] at the time that KRAS was thought to be undruggable. Tipifarnib has been tested in three phase 2 and one phase 3 clinical trials for PDAC. A phase 3 double-blind study [NCT00005648] [86] aimed to determine whether adding tipifarnib to gemcitabine could prolong survival in patients with advanced pancreatic cancer. The trial enrolled 688 patients, who were randomly assigned to two arms. The first arm received tipifarnib (200 mg twice daily via oral tablet) in combination with gemcitabine (1000 mg/m^2^ once a week for seven weeks, followed by one week off, then treatment resumed every three out of four weeks). The second arm received the same gemcitabine regimen with a placebo instead of tipifarnib. The study found that tipifarnib did not significantly increase overall survival, with a median OS of 193 days in the experimental arm versus 182 days in the control arm. Although tipifarnib met the toxicity parameters, it did not improve overall survival in pancreatic cancer patients. This redundancy—when farnesylation is inhibited, KRAS can undergo geranylgeranylation, allowing it to maintain it signaling function and tumorigenic activity—has limited the effectiveness of FTIs as single agents in treating KRAS-driven cancers [87].

### 5.10. Tyrosine Kinase Targeting Trials

Tyrosine kinases (TK) are involved in the signal transduction pathways that mediate communication between cells and their external environment. In PDAC, aberrant tyrosine kinase signaling can lead to uncontrolled cell proliferation, survival, and metastasis [88] (Figure 6). Other than EGFR which has been discussed earlier, drugs have been developed to target various TKs in treating chronic myelogenous leukemia, non-small cell lung cancer, HER2+ breast cancer, and GI tumors [89,90].

Imatinib mesylate, a BCR-ABL targeting agent, has now completed phase II of PDAC treatment. Masitinib, a novel c-KIT inhibitor, has completed two phase 3 trials for PDAC treatment. In trial [NCT00789633] [91], 353 chemotherapy-naive patients with inoperable PDAC were randomized to receive either gemcitabine (1000 mg/m^2^) with Masitinib (9 mg/kg/day) or gemcitabine with a placebo. The results showed no significant difference in median OS between the two treatment arms for the overall population, with 7.7 months for the Masitinib plus gemcitabine group and 7.1 months for the placebo plus gemcitabine group. Further analysis found that a subgroup with overexpression of acyl-CoA oxidase-1 (ACOX1) in blood had a median OS of 11.7 months (HR = 0.23, 95% CI [0.10; 0.51], *p* = 0.001), and the other subgroup with baseline pain intensity (VAS > 20 mm) had a median OS of 8.0 months (HR = 0.62, 95% CI [0.43; 0.89], *p* = 0.012) when treated with Masitinib, compared with a median OS of 5.5 months on single-agent gemcitabine. These findings suggest the potential for masitinib plus gemcitabine in PDAC patients with ACOX1 overexpression or baseline pain, warranting a confirmatory study to support its use.

Bruton’s tyrosine kinase (BTK) is a prominent B cell kinase implicated in the development of regulatory B cells. Targeting this kinase may prove to be an effective method to modulate the TME of PDAC towards a more immunologically active environment [92,93]. A phase 3 trial [NCT02436668] [94] utilized ibrutinib, a BTK inhibitor, in combination with nab-paclitaxel and gemcitabine. This study included 424 participants with stage IV PDAC, segregated into two arms. The ibrutinib arm contained 211 participants who received a daily dose of 560 mg of ibrutinib via oral tablet. The placebo arm, with 213 participants, received standard treatment alone. Both arms received 125 mg/m^2^ of nab-paclitaxel and 1000 mg/m^2^ of gemcitabine. Unfortunately, the trial did not result in a significant increase in OS, with the median OS for the ibrutinib arm at 9.7 months compared to 10.8 months for the placebo arm.

Dasatinib is a multi-targeting TKI agent most commonly used to treat various subtypes of leukemias [95]. Dasatinib was used in a phase II trial [NCT00474812] [96] to determine the impact dasatinib has on overall survival and objective response rate. This trial had 51 participants who received 100 mg of dasatinib orally, twice daily. This dosage was later reduced to 70 mg, twice daily, due to dose-related toxicity. The median OS for this group was 4.7 months, suggesting that dasatinib, as a single agent, is not sufficient to treat PDAC. It also proved to be inefficient in significantly prolonging OS when added to mFOLFOX6 [NCT01652976] [97] with a median OS of 10.6 months. Another phase 2 trial [NCT01395017] [98] also concluded that the addition of dasatinib to gemcitabine does not prolong survival, and the toxicity burden was a higher risk compared to the potential benefits.

Protein kinase C (PKC) inhibitors such as 7-hydroxystaurosporine and Bryostatin 1 have completed phase 2 trials. The PKCβ inhibitor Enzastaurin has also completed phase 2. Non-specific tyrosine kinase inhibitors are broadly used for multiple cancers but often come with significant side effects. Sunitinib has completed three phase 2 trials. Targeting non-receptor tyrosine kinases, often simply referred to as tyrosine kinases, is the most common strategy in this class, with eight phase 2 trials completed. This class includes drugs such as dasatinib (n = 4) and sorafenib tosylate (n = 4). The serine protease urokinase plays significant roles in cancer progression, tissue remodeling, and cellular migration. WX-671, a urokinase inhibitor, completed a phase 2 clinical trial in 2010. Other drugs within the kinase inhibitor class include saracatinib, a src kinase inhibitor, and galunisertib, a TGF-β receptor kinase inhibitor, both of which have completed phase 2 trials.

VEGF receptors play a crucial role in angiogenesis, the process by which new blood vessels form, which is essential for tumor growth and metastasis [99]. By inhibiting these receptors, VEGFR targeting drugs aim to starve the tumor of its blood supply, thereby inhibiting its growth and spread. PDAC is characterized by a dense stromal component and poor vascularization. Despite this, the tumor promotes angiogenesis to support its growth and metastatic potential. VEGFR targeting drugs have been in 3 phase 2 and 2 phase 3 trials. In one of the phase 3 trials [NCT00471146] [100], 632 patients were randomized to receive gemcitabine with either axitinib or a placebo. Gemcitabine was administered at 1000 mg/m^2^ intravenously on days 1, 8, and 15 of a 28-day cycle, while axitinib or placebo was given orally twice daily, starting at 5 mg and titrated up to 10 mg if tolerated. The median overall survival was 8.5 months for the axitinib group and 8.3 months for the placebo group. The trials concluded that the addition of axitinib to gemcitabine did not improve overall survival in advanced pancreatic cancer. This outcome suggests that targeting VEGF signaling is ineffective for PDAC.

### 5.11. Metabolism Associated Targeting Agents

**Figure 7:**
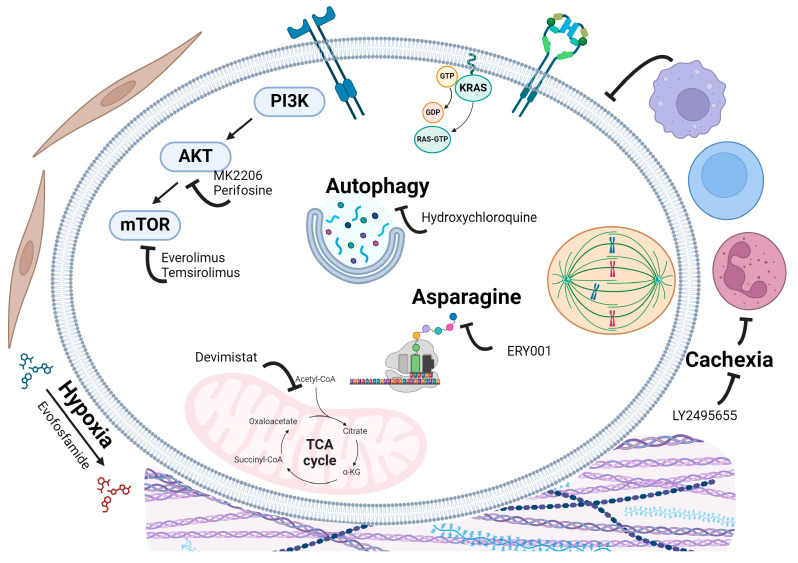
Targeting metabolic pathways. (Created with BioRender.com).

Cancer cells exhibit alterations in their metabolic pathways compared to normal cells, enabling them to sustain higher proliferative rates and adapt to the harsh tumor microenvironment (TME) [101]. These unique metabolic pathways, including the mTOR pathway and autophagy, are promising therapeutic targets due to the specific metabolic dependencies and vulnerabilities they create in tumor cells [102,103] (Figure 7). The KRAS mutation activates numerous proteins, including PI3K, which subsequently activates AKT, leading to mTOR activation. The mTOR pathway results in the activation of various proteins that alter cellular metabolism, proliferation, immune response, autophagy, and cell growth. Inhibition of these integrated pathways can potentially reduce signaling that promotes cell survival, proliferation, and metabolism.

### 5.12. Nutrient-Dependency Targeting Agents

Within the highly hypoxic and nutrient-deprived environment of PDAC, targeting unique metabolic dependencies has been a focus in a phase 3 trial [NCT03504423] [104]. This trial aimed to target the tricarboxylic acid (TCA) cycle, specifically the pyruvate dehydrogenase and α-ketoglutarate dehydrogenase complexes, using CPI-613 (Devimistat). By inhibiting these key carbon entry points, the study sought to determine whether adding CPI-613 could improve overall survival (OS) in patients, given PDAC’s reliance on mitochondrial function. A total of 528 patients were enrolled and divided into two arms. The first arm received FOLFIRINOX combined with 500 mg/m^2^ of CPI-613 via IV injection on days 1 and 3 of a 14-day cycle, while the second arm received FOLFIRINOX alone. The median OS was comparable between the two groups, with the CPI-613 group having a median OS of 11.1 months and the FOLFIRINOX-alone group having a median OS of 11.73 months. PFS was similar, with the CPI-613 group having a PFS of 7.82 months and the FOLFIRINOX group a PFS of 7.98 months. The lack of significant clinical efficacy in this trial may reflect tumor-intrinsic and extrinsic factors, indicating that more research is needed to identify patient populations that could benefit from this treatment.

### 5.13. mTOR Associated Targeting Agents

mTOR-targeting agents such as everolimus and temsirolimus have completed phase 2 trials for PDAC. In a phase 2 trial [NCT00409292] [105] evaluating RAD001 (everolimus) for patients with gemcitabine-refractory, metastatic pancreatic cancer. Thirty-three patients received RAD001 at 10 mg daily and were monitored for toxicity, treatment response, and survival. The treatment was well-tolerated. Median progression-free survival was 1.8 months, and overall survival was 4.5 months. One patient showed a biochemical response with a significant reduction in serum CA19-9 levels. Despite good tolerability, RAD001 as a single agent showed minimal clinical activity in this patient population. Future research should explore mTOR inhibitors in combination with other treatments or target other components of the PI3K/Akt/mTOR pathway.

No study result can be found for three phase 2 trials targeting AKT alone. A phase 2 trial [NCT01658943] [106] tested selumetinib, a MEK inhibitor, and MK2206, an AKT inhibitor with 137 participants. This two-arm study compared selumetinib + MK2206 with modified FOLFOX by evaluating their respective overall survival. The modified FOLFOX arm had a median OS of 6.7 months whereas the selumetinib + MK2206 arm had a 3.9-month median OS. Although the experimental arm failed to improve overall survival in PDAC patients, the results further emphasize the importance of publishing results to provide information to researchers and the public regarding investigations and corresponding results. This would increase awareness of previous studies while also allowing for investigation concerning why these drugs fail.

### 5.14. Autophagy

Autophagy, the cellular process of degrading and recycling intracellular components, is a crucial target for cancer treatment due to its role in helping cancer cells survive under stress conditions [107]. Cancer cells often face metabolic stress from rapid proliferation, hypoxia, and nutrient deprivation, and autophagy provides an alternative energy source that aids in their survival [108]. This process can also contribute to therapeutic resistance by protecting cancer cells from the cytotoxic effects of chemotherapy and radiation [109]. By inhibiting autophagy, these protective mechanisms are disrupted, potentially enhancing treatment efficacy and overcoming resistance.

Two phase 2 trials targeting autophagy have been completed. The [NCT01978184] [110] randomized trial assessed gemcitabine/nab-paclitaxel with or without hydroxychloroquine (HCQ). The study used Fisher’s exact test and log-rank test to assess the response and survival outcomes related to SMAD4 status. Fifty-two patients receiving HCQ with neoadjuvant chemotherapy were studied, with 25 patients (48%) showing SMAD4 loss. Among these, 76% with SMAD4 loss achieved a histopathologic response of grade ≥2A, compared to only 37% with SMAD4 intact (*p* = 0.006). Despite SMAD4 loss typically being associated with worse outcomes, this study found no significant difference in median overall survival between HCQ-treated patients with SMAD4 loss (34.43 months) and those with SMAD4 intact (27.27 months, *p* = 0.18). The addition of HCQ to neoadjuvant chemotherapy may improve treatment response in patients with PDAC who have SMAD4 loss, suggesting that HCQ could potentially counteract the therapeutic resistance induced by autophagy upregulation. Further research is warranted to explore the interplay between SMAD4, autophagy, and treatment outcomes in PDAC.

### 5.15. Asparagine

Cancer cell metabolism is highly dysregulated and often deviates from more efficient energy production pathways to pursue alternative methods of energy production [111]. This reprogrammed metabolism within cancer cells provides opportunities for therapeutic intervention. One promising target within cancer cell metabolism is asparagine, a non-essential amino acid involved in canonical protein synthesis and many downstream signaling pathways. The rationale for targeting asparagine in solid and blood cancers lies in the limited bioavailability of glutamine within the TME [112,113]. By restricting asparagine, it may be possible to prevent aberrant signaling and inhibit cancer cells’ adaptation to the lack of nutrient availability, thereby impeding their survival and proliferation.

Asparagine is typically targeted with asparaginase, an enzyme that degrades asparagine, and is commonly used in chemotherapy treatments for acute lymphoblastic leukemia. One phase 2 and one phase 3 trial have explored the use of asparaginase in PDAC treatment, where asparaginase was delivered by red blood cells. The phase 2 trial [NCT02195180] [114] incorporated asparaginase (ERY001) into the standard of care. In this trial, 141 participants were divided into two arms: the control arm (n = 46), which received standard chemotherapy alone (FOLFOX + gemcitabine), and the experimental arm (n = 95), which received standard chemotherapy plus asparaginase. The median overall survival (OS) for the experimental arm was 6.0 months, compared to 4.4 months for the control arm. This trial confirmed the safety of combining asparaginase with chemotherapy and demonstrated an increase in median OS. The phase 3 trial [NCT03665441] [115] utilized a larger cohort, incorporating asparaginase (eryaspase) into chemotherapy. A total of 512 participants were divided into two arms: the experimental arm (n = 255) and the control arm (n = 257). The experimental arm received eryaspase with standard chemotherapy, while the control arm received standard chemotherapy alone. The median OS for the experimental arm was 7.5 months, compared to 6.7 months for the control arm. Although the addition of asparaginase to the standard of care resulted in a non-significant increase in median OS, it did show encouraging survival outcomes and warrants further investigation.

### 5.16. Cachexia

Cancer cachexia is a complex syndrome characterized by severe body weight, fat, and muscle loss and is often accompanied by anorexia and metabolic changes [116,117,118]. 80% of PDAC patients have cachexia, the highest among all cancers. The exact mechanisms of cancer cachexia are not entirely understood, but it involves a combination of factors such as systemic inflammation, metabolic abnormalities, and the tumor’s direct effects. Cytokines and other inflammatory mediators released by the tumor and the host’s immune response play a significant role in driving the metabolic alterations that lead to muscle wasting and fat loss [119,120,121]. Cachexia not only contributes to a decreased quality of life, but also negatively impacts the efficacy of cancer treatments and overall survival.

[NCT01505530] [122] is a clinical trial that utilizes the drug LY2495655, a monoclonal antibody targeting myostatin. Inhibition of myostatin has been demonstrated to prevent myostatin-induced satellite cell activation inhibition [123]. As myostatin inhibits satellite cell activation, this ultimately prevents muscle hypertrophy. This trial has three major groups that consist of LY2495655, placebo group, and standard of care chemotherapy group. The study has 125 participants isolated into three arms of this study involving arm 1300 mg LY2495655 in addition to chemotherapy (n = 41), arm 2100 mg LY2495655 in addition to chemotherapy (n = 43), and arm 3 placebo group with chemotherapy alone (n = 41). The OS for the LY2495655 groups was not significantly extended compared with the placebo group, as the median OS for arm 1 was 8.02 months, the median OS for arm 2 was 9.82 months, and median OS for arm 3 was 10.45 months. Although the OS was not increased in the treatment group, there was a discernible, but not statistically significant, difference in lean mass and functional evaluations. It was noticed that those who displayed precachexia (<5% weight loss) had greater benefits from LY2495655 treatment compared to those who displayed signs of cachexia (≥5% weight loss).

### 5.17. Hypoxia

PDAC exhibits poor vascularization and limited oxygen supply, creating a severely hypoxic environment that healthy cells cannot withstand. This unique characteristic led to the development of TH-302, a hypoxia-activated prodrug designed to release the DNA-alkylating agent bromo-isophosphoramide mustard specifically within the hypoxic regions of tumors. The selective activation of this prodrug allows for targeted delivery of the DNA-damaging agent to the tumor. A phase 2 trial [NCT01144455] [124] demonstrated an increase in median overall survival (OS) with TH-302, leading to the MAESTRO phase 3 study [NCT01746979] [125], which aimed to assess the clinical efficacy of TH-302 (Evofosfamide) in a larger cohort. In this study, 693 patients with locally advanced, unresectable, and/or metastatic pancreatic adenocarcinoma were divided into two groups. The first group received gemcitabine (1000 mg/m^2^) on days 1, 8, and 15 of every 28-day cycle, along with TH-302 (340 mg/m^2^) on the same regimen. The control group received gemcitabine alone with a placebo (5% dextrose). The TH-302 group exhibited a significantly higher median PFS of 5.5 months compared to 3.7 months in the control group (*p* = 0.004). Median OS was also higher in the TH-302 group, at 8.9 months, compared to 7.6 months in the control group, though this difference was not statistically significant (*p* = 0.059). Despite the lack of significant improvement in median OS, TH-302 significantly increased PFS and showed a larger objective response rate. The safety profile of the drugs was deemed acceptable. These findings suggest that TH-302 could be a valuable addition to gemcitabine therapy to enhance PFS, and the hypoxia-activated prodrug strategy may hold promise for other drugs, potentially improving efficacy and reducing toxicity.

### 5.18. Immune Modulating Drugs

**Figure 8:**
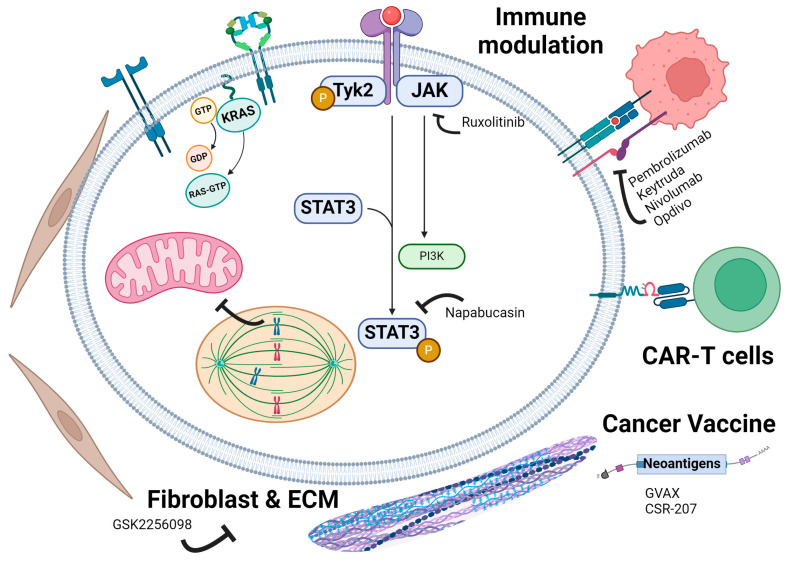
Targeting immune modulating pathways. (Created with BioRender.com).

The immune cells recognize and eliminate cancer cells through immune surveillance, thus playing a crucial role in cancer development and treatment. Tumors can evade immune detection through different mechanisms. Various treatments have been developed to modulate the immune system to destroy cancer cells (Figure 8).

The JAK/STAT signaling pathway plays a significant role in tumor progression and immune evasion in PDAC [126] (Figure 8). The pathway involves the Janus kinases (JAK1, JAK2, JAK3, and TYK2) and the signal transducers and activators of transcription (STATs), which transmit extracellular signals from cytokines and growth factors to the cell nucleus, resulting in the transcription of genes involved in proliferation, survival, and immune responses [127]. In PDAC, the JAK/STAT pathway is frequently activated, leading to increased tumor growth, survival, and resistance to apoptosis. The activation of this pathway can occur through various mechanisms, including mutations, autocrine or paracrine cytokine signaling, and interaction with the tumor microenvironment. Notably, STAT3 is often hyperactivated in PDAC, promoting oncogenic processes such as inflammation, angiogenesis, and metastasis [128].

In a phase 2 clinical trial [NCT01423604] [129] using ruxolitinib, a JAK1/2 kinase inhibitor, 136 participants with high-grade PDAC were separated into two arms. The first arm (n = 64) received 15 mg of ruxolitinib twice daily and 1000 mg/m^2^ of capecitabine twice daily, while the second arm received capecitabine alone. The median overall survival for the first arm was 136.5 days with a progression-free survival rate of 51 days. The median overall survival for the second arm was 129.5 days with a progression-free survival rate of 46 days. The addition of JAK1/2 inhibitors to capecitabine did not significantly increase survival but may have the potential to prolong survival when added to other treatment cocktails.

Another strategy has been to target STAT3, a highly active and pivotal transcription factor that is upregulated in PDAC. A phase III clinical trial [NCT02993731] [130] targeted STAT3 with the small molecule inhibitor napabucasin. In this trial, 1134 participants were randomized into two arms. The first arm (n = 565) received napabucasin with nab-paclitaxel and gemcitabine, while the second arm (n = 569) received nab-paclitaxel and gemcitabine alone. Nab-paclitaxel and gemcitabine were administered once weekly for three weeks in a four-week period, and napabucasin was administered twice daily via oral tablet. The median overall survival (OS) for the first arm was 11.43 months. The median OS for the second arm was 11.73 months. This trial determined that the STAT3 inhibitor napabucasin was not a beneficial additive to the standard treatment regimen and did not significantly extend overall survival.

Immune modulating drugs, also known as immunotherapies, have revolutionized cancer treatment by harnessing the body’s immune system to recognize and destroy cancer cells. These therapies work by enhancing the immune response against tumors, offering potential long-term control and remission of the disease. The primary strategies include checkpoint inhibitors [131,132,133], cytokines, cancer vaccines, and adoptive cell transfer therapies [134,135,136,137]. Checkpoint inhibitors such as pembrolizumab (Keytruda) and nivolumab (Opdivo) target proteins like PD-1/PD-L1 and CTLA-4, which normally help keep immune responses in check but are exploited by cancer cells to avoid attack. However, their efficacy in PDAC has been limited due to the tumor’s immunosuppressive microenvironment. Cytokine therapies, including CXCR4 antagonist, aim to enhance the immune system’s ability to attack cancer cells [138]. Research into cytokine combinations and delivery methods is ongoing to improve their effectiveness in PDAC.

The success of Immune modulating drugs in PDAC requires overcoming the dense stromal tissue and immunosuppressive microenvironment. A phase 2 trial [NCT02428270] tested GSK2256098, a very selective inhibitor of FAK kinase. FAK has been shown to suppress antigen processing and presentation to promote immune evasion in pancreatic cancer [139]. FAK inhibition also reprograms stromal cells, primes tumor immunity and unlocks responsiveness to checkpoint immunotherapy [140]. Combination therapies, involving immune checkpoint inhibitors, cytokines, and traditional treatments like chemotherapy and radiation, are being explored to enhance efficacy. Drugs targeting stromal tissues were also tested in PDAC. These combined approaches hold promise for improving outcomes in PDAC treatment.

Cancer vaccines aim to stimulate the immune system to target cancer-specific antigens [141,142]. GVAX, a granulocyte-macrophage colony-stimulating factor (GM-CSF) secreting pancreatic cancer vaccine, and CRS-207 CRS-207, a live-attenuated Listeria monocytogenes expressing mesothelin, are being studied for their potential to boost immune responses in PDAC [143,144]. Adoptive cell transfer therapies, such as chimeric antigen receptor (CAR) T-cell therapy, involve engineering a patient’s T cells to better recognize and attack PDAC cells. These therapies are still in the experimental stages for PDAC.

### 5.19. Summary

Nearly one-third of the 208 completed trials we analyzed focus on DNA and microtubule targets. The microtubule-targeting agent Nab-Paclitaxel remains a standard treatment in PDAC, with no superior alternatives. Similarly, other than cisplatin, no other DNA-damaging agents or DNA synthesis inhibitors have proven more effective than gemcitabine and FOLFIRINOX. While EGFR and TK inhibitors have not shown significant efficacy, they continue to be evaluated. Promising results are emerging from trials involving immune-modulating drugs, which will be discussed in the next section.

## 6. Glance on Ongoing Trials

The substantial number of ongoing clinical trials for PDAC underscores the immense effort to combat this particularly aggressive and lethal cancer. The complexity of its biology, involving numerous genetic and molecular pathways, requires extensive research. Moreover, PDAC’s notorious resistance to conventional treatments necessitates innovative approaches, including combination therapies, targeted treatments, and immunotherapies. Advances in cancer biology and technology further contribute to the proliferation of trials, each exploring different mechanisms of action. Additionally, the heterogeneity of PDAC means that diverse patient subgroups respond differently to treatments, highlighting the need for the identification of biomarkers to better stratify patients for treatment. Increased funding and regulatory support have facilitated this expansive research landscape. Collectively, these factors highlight the urgent need for multifaceted approaches to improve outcomes for PDAC patients.

**Figure 9:**
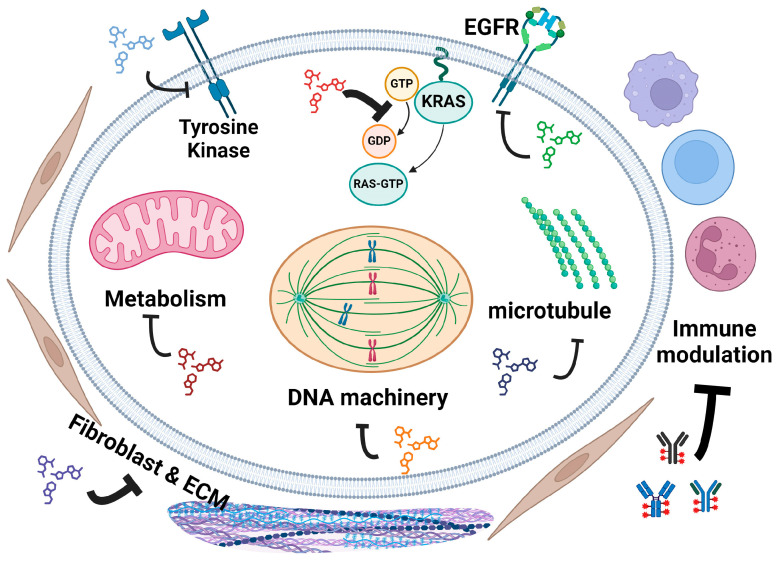
Summary of ongoing trials. The thickness of the inhibitory bar indicates the number of trials. (Created with BioRender.com).

The landscape of ongoing clinical trials for PDAC is robust and dynamic, reflecting the urgent need for effective treatments for this aggressive cancer (Figure 9) (Supplemental Table S4). Currently, there are 1471 active trials, including the study statuses of “not yet recruiting”, “recruiting”, and “active but not recruiting”. Drug interventions dominate these efforts, accounting for 711 trials. While more than half are in early-stage investigation, 339 of these trials are in phase 2 or higher, indicating a substantial focus on more advanced stages of clinical testing. Among these, 256 trials are dedicated solely to PDAC, highlighting the concentrated effort to find targeted therapies for this specific cancer type. Notably, 78 of these ongoing trials continue to explore dosage optimization, combinations, or new formulations of established drugs like gemcitabine (G), fluorouracil (F), and paclitaxel (P). Furthermore, 155 trials which we will discuss below are investigating unique drugs or novel combinations with G/F/P, aiming to enhance therapeutic efficacy and overcome resistance mechanisms. This extensive array of trials underscores the complex and multifaceted approach needed to tackle PDAC, involving a blend of refining existing treatments and pioneering new therapeutic avenues.

### 6.1. Immunotherapy Trials

The landscape of ongoing trials for PDAC highlights the substantial focus and investment in leveraging the immune system to combat this aggressive cancer. Despite the promise shown by immunotherapies in other cancers, PDAC has proven particularly challenging, with limited success to date. Among the 155 trials utilizing unique or combination drugs, 78 are testing immunotherapy alone or in combination with standard chemotherapies or other drugs. The majority of these, 63 trials, investigate checkpoint inhibitors targeting the PD-1/PD-L1 or CTLA-4 axis. This focus on PD-1/PD-L1 reflects the broad interest in checkpoint inhibitors as a therapeutic strategy.

However, there is also a noteworthy exploration of alternative immunotherapeutic targets, either alone or in combination with immune checkpoint inhibitors. Other targets being tested with checkpoint blockade include CD38, CD40, CD73, IL-1β, and CXCL12. 13 trials do not involve PD-1 blockade, but instead investigate other pathways and mechanisms. These include trials targeting IL-2, CSF-R, IL-1R, IL-2R, IL-6R, adoptive NK cells, and various cancer vaccines. These diverse approaches highlight the multifaceted efforts to harness the immune system against PDAC and indicate a broad-based strategy to identify effective immunotherapeutic interventions. The extensive range of targets and strategies being investigated underscores the complexity of PDAC and the need for innovative solutions to improve patient outcomes.

### 6.2. KRAS Inhibitors

Oncogenic mutations of KRAS occur in over 90% of PDAC cases. These mutations, primarily point mutations at codon 12 leading to substitutions such as G12D, G12V, G12C, and G12R, result in the KRAS protein being locked in an active GTP-bound state. This constitutive activation drives downstream signaling pathways, particularly the RAF-MEK-ERK and PI3K-AKT pathways, promoting uncontrolled cell proliferation and resistance to apoptosis. Although farnesyltransferase inhibitors, which block KRAS post-translational modification, showed no clinical effect, targeting KRAS directly is still highly attractive and has remained challenging due to its high affinity for GTP/GDP and the absence of suitable binding sites for small-molecule inhibitors. However, recent advances have led to the development of multiple promising KRAS inhibitors [145,146,147], including mutation-specific inhibitors targeting G12C [148,149,150] or G12D mutants [151,152]. Currently, only one phase 2 trial [NCT06008288] is in the recruiting stage to test the G12C-specific inhibitor JAB-21822, with most trials testing KRAS inhibitors still in early phases (supplemental Table S3). Recently, pan-KRAS inhibitors have been developed and tested in preclinical models [153,154,155]. Two phase 1 trials, [NCT06128551] and [NCT06078800] are recruiting patients to evaluate these inhibitors.

### 6.3. Trials Targeting Fibrosis

Targeting fibrosis in PDAC is crucial due to its significant impact on treatment efficacy. The dense fibrotic tissue characteristic of PDAC acts as a physical barrier that limits the penetration of therapeutic drugs, thereby reducing their effectiveness [156]. This fibrotic stroma also obstructs the delivery of essential nutrients and oxygen, creating a hypoxic environment that promotes aggressive tumor behavior and resistance to conventional therapies. Additionally, fibrosis impedes immune cell infiltration, diminishing the effectiveness of immunotherapies. By targeting fibrosis, the tumor microenvironment (TME) can be remodeled to support better drug delivery and immune response, ultimately enhancing the efficacy of combined treatment strategies such as chemotherapy, targeted therapy, and immunotherapy [157]. Therefore, addressing fibrosis is essential for improving treatment outcomes in PDAC.

There are 7 active trials focused on targeting ECM and TME components. Among these, the FAK inhibitor, which is aimed at disrupting the fibrotic stroma, will be tested in a trial that incorporates PD-1. Heparin, retinoic acid, and vitamin D2 represent promising therapeutic agents for targeting TME, enhancing immune responses, and directly inhibiting cancer cell growth and metastasis. There are more early phase trials testing new drugs that can modify TME, reflecting a multifaceted approach to overcoming the dense fibrotic barriers in PDAC and enhancing the effectiveness of cancer therapies.

### 6.4. DNA-Damaging and Repair-Targeting Agents

In addition to the current front-line DNA-damaging agents as standard treatments for PDAC, a large number of completed trials have tested novel drugs targeting DNA replication, while additional agents continue to be explored in ongoing trials. Currently, 22 trials are focused on targeting various aspects of DNA synthesis and homeostasis, including DNA-damaging agents, DNA repair inhibitors, and epigenetic regulators. For example, TAS 102 combines trifluridine, which is incorporated into DNA causing damage, and thymidine phosphorylase inhibitors (TPIs), which inhibit a critical enzyme in the pyrimidine pathway, thereby increasing the bioavailability of trifluridine. Other DNA-targeting agents in clinical trials include cisplatin, cyclophosphamide, and chlorambucil. Eight trials are currently testing PARP inhibitors in combination with chemotherapies and immune checkpoint blockade agents. Additionally, two phase II trials are currently evaluating DNA methylation inhibitors with the drugs decitabine and azacitidine. More drugs in this class are being tested in early-phase trials for safety. Thus, targeting DNA and DNA repair pathways remains an attractive strategy for PDAC treatment.

### 6.5. Metabolism

Targeting metabolism remains an attractive strategy for ongoing cancer trials due to the unique metabolic dependencies of PDAC cells, which support their rapid growth and survival in the TME. Metabolic targeting can also help overcome resistance mechanisms associated with traditional therapies, offering a new route to enhance treatment efficacy. Combining metabolic inhibitors with other therapeutic agents, such as chemotherapies or immunotherapies, has led to the proposal of more effective treatment regimens. The specificity of certain metabolic pathways in cancer cells compared to normal cells allows for selective targeting, minimizing damage to healthy tissues and reducing side effects. Advances in cancer metabolism have identified new targets, such as glutamine metabolism and fatty acid synthesis, providing fresh opportunities for drug development. Hydroxychloroquine, currently being investigated for its role in autophagy inhibition, is in 3 ongoing phase 2 trials in combination with other agents. Anamorelin hydrochloride, a ghrelin receptor agonist, is being tested in an ongoing phase 2 trial for combating cachexia.

## 7. Conclusions

Collectively, this review describes all categories of clinical trials for PDAC available on ClinicalTrials.gov, including temporal analysis of trial frequency, intervention type, percentage of exploration, and an in-depth description of completed and ongoing drug clinical trials. This review has shown that of more than 4000 clinical trials, drug trials dominate when stratifying all the trials by intervention type, accounting for 49.6% of all cases. Drug trials, spanning from the late 1970s to the present day, have shown little to no efficacy from the tested regimens, while the most common drugs currently used to treat PDAC were established in the 1970s, indicating minimal advancements since then. Despite the lack of efficacy of novel therapeutics employed to treat PDAC, researchers and clinicians have not been deterred. There are currently more clinical trials than ever before, incorporating novel therapeutics, repurposed drugs, and combination therapies. Ongoing clinical trials are now targeting not only the cancer itself but also the microenvironment, structures, and immune cells to increase the efficacy and potency of both novel and established drugs to combat PDAC.

## 8. Perspective

### 8.1. Improvements for Website Navigation

Researchers have navigated ClinicalTrials.gov for various cancers and have identified shortcomings that need improvement [158,159,160,161,162,163]. During the collection and analysis of PDAC clinical trials from ClinicalTrials.gov, we also found several major areas in need of improvement. Issues regarding the search for clinical trials, intervention types, categorization, and results are largely inconsistent or incomplete, rendering the website and search unreliable. For instance, a search for “PDAC—Pancreatic Ductal Adenocarcinoma” resulted in 432 studies, while a search for “pancreatic cancer” yielded over 4000 studies. There are many names under “Condition” for PDAC, including “Pancreatic Neoplasm”, “Cancer of the Pancreatic Head”, “Adenocarcinoma of the Pancreas”, and “Duct Cell Adenocarcinoma of the Pancreas”. A standardized dropdown menu for consistent condition terminology for every study recorded on ClinicalTrials.gov would be beneficial.

Interventions are categorized inconsistently. Antibodies, for example, are considered both “Biologicals” and “Drugs”, as is adoptive cell transfer, complicating analysis. One major limitation of the “Intervention” category is that it lists drug names without providing any information about their targets and functions. We have compiled a table listing drugs with their targets, and it would be helpful to add a column specifying the target of each drug. Exposure to novel therapeutics and interventions not broadly known to the public or researchers unfamiliar with drug names or novel therapeutic targets could be improved by including options to search by therapeutic target or mode of intervention.

Additionally, providing categories that are easily accessible and predesignated would reduce the impact of filtering on results. Adding a dropdown menu for the purpose of the study, such as “dosage modification”, “drug delivery route”, “unique drug”, or “combination with standard chemo”, would also be helpful. Lastly, updates to clinical trials are scarce on the website. The column titled “Results Posted” often does not reflect the most recent advances made within the trial. Some indicate “Yes” for study results but lack clear results or listed publications. Many trials that claimed “no results” on the website were found to have publications regarding methodologies and results, which could not be discovered on the website without an independent literature search. Addressing these issues could not only improve the fidelity and reliability of ClinicalTrials.gov but also enhance ongoing and future PDAC research by integrating prior discoveries, thereby accelerating the exploration of promising combination therapies.

### 8.2. Current Challenges and Future Opportunities

PDAC is one of the most aggressive and lethal forms of cancer, presenting numerous challenges and opportunities for treatment. One of the primary challenges is the late diagnosis of PDAC. Often, PDAC is diagnosed at an advanced stage due to its asymptomatic nature in early stages as well as no screening tests. This late diagnosis significantly limits treatment options and reduces survival rates. Therefore, there is a significant need to develop and implement early detection methods. This can include the use of circulating biomarkers as well as improved radiographic imaging or the use of radiomics to better detect early lesions. Genetic and biomarker screening also offer significant opportunities for PDAC treatment. Screening can identify individuals at high risk for PDAC and facilitate early intervention. Additionally, the personalized medicine approach enables the development of treatment strategies specifically tailored to the genetic profile of both the tumor and the patient. Technological advancements in imaging, liquid biopsies, and genomic sequencing can enhance early detection, monitoring, and treatment planning for PDAC patients. These tools can provide real-time insights into tumor dynamics and treatment response.

Artificial intelligence (AI) and machine learning (ML) hold significant potential for advancing the diagnosis, treatment, and management of PDAC [164,165]. Cao et al. developed a new deep learning approach named PANDA (pancreatic cancer detection with artificial intelligence) to accurately detect and classify pancreatic lesions using non-contrast CT scans. Trained on a dataset of 3208 patients from one center, PANDA achieved an area under the receiver operating characteristic curve (AUC) of 0.986–0.996 for lesion detection in a multicenter validation involving 6239 patients across ten centers. It achieved a sensitivity of 92.9% and specificity of 99.9% in a real-world validation of 20,530 patients, suggesting its potential as a new tool for large-scale pancreatic cancer screening [166].

Improving treatment options for PDAC following diagnosis remains a critical challenge, particularly since most cases are identified at advanced stages. While there have been advancements in cytotoxic chemotherapy, there is still considerable work needed to enhance systemic therapies. Targeted therapies offer promising potential, especially with the identification of key genetic mutations and pathways in PDAC, such as KRAS, SMAD4, and BRCA1. These discoveries enable the development of treatments that specifically address the molecular drivers of the disease, potentially improving patient outcomes. Additionally, the increased identification of actionable mutations in PDAC could open new avenues for treating specific patient subsets.

Chemoresistance is another major hurdle in the treatment of PDAC. PDAC cells often have intrinsic or acquired resistance to chemotherapy, reducing the long-term effectiveness of treatment regimens. However, combining traditional chemotherapies with novel agents that target resistance mechanisms, such as PARP inhibitors or metabolic inhibitors, can potentially overcome chemoresistance. Despite numerous clinical trials, few new therapies have shown significant improvement in survival rates for PDAC patients. Continued research into combinatorial therapies, including the use of immunotherapies, targeted therapies, and personalized medicine approaches, may yield more effective treatment options.

There are several promising areas that can be expanded on to increase our understanding of the disease process and can serve as potential targets for therapy. A deeper understanding of the metabolic pathways that are uniquely altered in PDAC is essential. This includes pathways like glycolysis, glutaminolysis, and lipid metabolism, which cancer cells rely on for rapid growth and survival in the tumor microenvironment. Identifying key metabolic enzymes and transporters that are overexpressed in PDAC can provide specific targets for drug development. Combining metabolic inhibitors with existing chemotherapies or immunotherapies can enhance treatment efficacy and overcome resistance mechanisms. Moreover, personalized medicine approaches, using metabolic profiling to tailor treatments to the specific metabolic vulnerabilities of an individual’s tumor, could improve outcomes. Advanced technologies, such as CRISPR and RNA interference, can be utilized to validate metabolic targets and screen for novel inhibitors. Finally, integrating robust biomarker development to monitor treatment response and metabolic adaptation can guide therapeutic adjustments, ensuring sustained efficacy and minimizing side effects.

Targeting cachexia, though not directly affecting cancer cells, can significantly improve cancer outcomes by enhancing patients’ overall health, strength, and quality of life, thereby making them better able to tolerate and respond to cancer treatments. By addressing the muscle wasting and weight loss associated with cachexia, patients can maintain their physical function and independence, which is crucial for enduring the rigors of chemotherapy, radiation, or surgery. Improved nutritional status and reduced inflammation can boost the immune system, potentially enhancing the body’s natural ability to combat cancer. Additionally, better-managed cachexia can reduce hospitalizations and complications, leading to a more continuous and effective cancer treatment regimen. Ultimately, by mitigating the debilitating effects of cachexia, patients have a better chance of completing their prescribed treatments, which can lead to improved survival rates and overall outcomes.

The aggressive biology of PDAC also poses a significant challenge. PDAC is characterized by rapid progression, making it difficult to treat with conventional therapies. Additionally, the dense fibrotic stroma surrounding PDAC tumors limits the delivery of therapeutic agents and immune cells to the tumor site, reducing the efficacy of treatments. Targeting the stroma and modifying the tumor microenvironment (TME) to enhance drug delivery and immune cell infiltration presents a promising therapeutic avenue. Agents that can modulate the TME or degrade the stroma may improve the efficacy of existing treatments.

The TME in PDAC displays a high presence of regulatory T cells, myeloid-derived suppressor cells, and tumor-associated macrophages, which collectively suppress effective anti-tumor immune responses. Additionally, PDAC tumors often exhibit low mutational burden, leading to fewer neoantigens that can be recognized by the immune system, making them less responsive to immune checkpoint inhibitors that have shown success in other cancer types. Despite these challenges, there are notable opportunities for advancing immune therapies in PDAC. Combination therapies that integrate immune checkpoint inhibitors with other treatments such as chemotherapy, radiation, or targeted therapies are being actively investigated to overcome the immunosuppressive TME. These combination approaches aim to enhance the efficacy of immune therapies by modifying the TME to be more conducive to immune activation. Additionally, advancements in personalized medicine, including the identification of specific biomarkers, can help tailor immune therapies to the unique characteristics of each patient’s tumor, potentially improving response rates.

Gene therapy for PDAC is an emerging field that aims to introduce genetic material into cells to either correct genetic defects, kill cancer cells, or enhance the immune response against the tumor. Innovative strategies such as adoptive cell therapies, including CAR-T cells and TCR-engineered T cells, are being explored. These approaches involve modifying patients’ own immune cells to better recognize and attack PDAC cells. Furthermore, novel immunomodulatory agents targeting pathways like the STING pathway, and oncolytic viruses designed to stimulate immune responses within the tumor, hold promise in transforming the treatment landscape for PDAC. In addition, pre-clinical development of a novel, non-viral gene therapy called CYL-02 has been shown to be safe and effective in rodent models. CYL-02 utilizes plasmid DNA containing genes for somatostatin receptor 2 (SSTR2), deoxycytidine kinase (DCK), and uridylate monophosphate kinase (UMK), which together aim to restore gene function in PDAC cells while also sensitizing the tumor to gemcitabine [167]. We anticipate that more innovative gene therapy strategies will emerge in the near future.

Lastly, targeting KRAS directly has historically been challenging due to the protein’s high affinity for GTP/GDP and the lack of suitable binding pockets for small molecules, making KRAS “undruggable” for many years. Recent advancements have led to the development of specific KRAS inhibitors [168,169], such as AMG-510 for KRASG12C [170] and MRTX1133 for KRASG12D [152,171,172]. Most recently, the pan-KRAS inhibitors [173,174] and the pan-RAS inhibitor RMC-7977 [154,155] have been tested in preclinical models, demonstrating the potential to treat various RAS-driven cancers, including PDAC. A related RAS(ON) multi-selective inhibitor, RMC-6236, is currently undergoing clinical evaluation, offering hope for more effective treatments against RAS-addicted cancers. However, like other drugs, resistance to KRAS inhibitors is expected, and the drug penetrance to the tumor TME is also a confounding factor for their efficacy. Future studies will focus on modifying the drugs to enhance delivery to cancer cells and targeting resistance mechanisms.

In summary, while PDAC treatment faces significant challenges due to the disease’s aggressive nature and late diagnosis, numerous opportunities exist through advancements in early detection, targeted therapies, immunotherapy, and combination treatments. Continued research and innovation are essential to improving outcomes for PDAC patients.

## Supplementary Materials

The following supporting information can be downloaded at: https://www.mdpi.com/article/10.3390/cancers16213564/s1, Table S1. Clinical Trials of Pancreatic Cancer from 1978-2024. Table S2. Drugs used in Trials. Table S3. Completed PDAC Trials (C: Combination, I: Immune, U: Unique, M: Method, G: Gemcitabine, F: FOLFIRINOX or components of FOLFIRINOX, P: Paclitaxel). Table S4. Ongoing PDAC Trials (C: Combination, I: Immune, U: Unique, M: Method, G: Gemcitabine, F: FOLFIRINOX or components of FOLFIRINOX, P: Paclitaxel).

## References

[1] Siegel R.L., Miller K.D., Wagle N.S., Jemal A. (2023). Cancer statistics, 2023. CA Cancer J. Clin..

[2] Halbrook C.J., Lyssiotis C.A., Pasca di Magliano M., Maitra A. (2023). Pancreatic cancer: Advances and challenges. Cell.

[3] Stoffel E.M., Brand R.E., Goggins M. (2023). Pancreatic Cancer: Changing Epidemiology and New Approaches to Risk Assessment, Early Detection, and Prevention. Gastroenterology.

[4] Park W., Chawla A., O’Reilly E.M. (2021). Pancreatic Cancer: A Review. JAMA.

[5] Golan T., Raitses-Gurevich M., Beller T., Carroll J., Brody J.R. (2023). Strategies for the Management of Patients with Pancreatic Cancer with PARP Inhibitors. Cancer Treat. Res..

[6] Keane F., O’Connor C.A., Park W., Seufferlein T., O’Reilly E.M. (2023). Pancreatic Cancer: BRCA Targeted Therapy and Beyond. Cancers.

[7] Kamarajah S.K., Burns W.R., Frankel T.L., Cho C.S., Nathan H. (2017). Validation of the American Joint Commission on Cancer (AJCC) 8th Edition Staging System for Patients with Pancreatic Adenocarcinoma: A Surveillance, Epidemiology and End Results (SEER) Analysis. Ann. Surg. Oncol..

[8] Amin M.B., Greene F.L., Edge S.B., Compton C.C., Gershenwald J.E., Brookland R.K., Meyer L., Gress D.M., Byrd D.R., Winchester D.P. (2017). The Eighth Edition AJCC Cancer Staging Manual: Continuing to build a bridge from a population-based to a more “personalized” approach to cancer staging. CA Cancer J. Clin..

[9] Che W.-Q., Li Y.-J., Tsang C.-K., Wang Y.-J., Chen Z., Wang X.-Y., Xu A.-D., Lyu J. (2023). How to use the Surveillance, Epidemiology, and End Results (SEER) data: Research design and methodology. Mil. Med. Res..

[10] Blackford A.L., Canto M.I., Dbouk M., Hruban R.H., Katona B.W., Chak A., Brand R.E., Syngal S., Farrell J., Kastrinos F. (2024). Pancreatic Cancer Surveillance and Survival of High-Risk Individuals. JAMA Oncol..

[11] Hansel D.E., Kern S.E., Hruban R.H. (2003). Molecular pathogenesis of pancreatic cancer. Annu. Rev. Genom. Hum. Genet..

[12] Martínez-Jiménez F., Movasati A., Brunner S.R., Nguyen L., Priestley P., Cuppen E., Van Hoeck A. (2023). Pan-cancer whole-genome comparison of primary and metastatic solid tumours. Nature.

[13] Waddell N., Pajic M., Patch A.-M., Chang D.K., Kassahn K.S., Bailey P., Johns A.L., Miller D., Nones K., Quek K. (2015). Whole genomes redefine the mutational landscape of pancreatic cancer. Nature.

[14] Witkiewicz A.K., McMillan E.A., Balaji U., Baek G., Lin W.-C., Mansour J., Mollaee M., Wagner K.-U., Koduru P., Yopp A. (2015). Whole-exome sequencing of pancreatic cancer defines genetic diversity and therapeutic targets. Nat. Commun..

[15] Bailey P., Chang D.K., Nones K., Johns A.L., Patch A.-M., Gingras M.-C., Miller D.K., Christ A.N., Bruxner T.J.C., Quinn M.C. (2016). Genomic analyses identify molecular subtypes of pancreatic cancer. Nature.

[16] Roberts N.J., Norris A.L., Petersen G.M., Bondy M.L., Brand R., Gallinger S., Kurtz R.C., Olson S.H., Rustgi A.K., Schwartz A.G. (2016). Whole Genome Sequencing Defines the Genetic Heterogeneity of Familial Pancreatic Cancer. Cancer Discov..

[17] Cancer Genome Atlas Research Network (2017). Electronic address: Andrew_aguirre@dfci.harvard.edu; Cancer Genome Atlas Research Network Integrated Genomic Characterization of Pancreatic Ductal Adenocarcinoma. Cancer Cell.

[18] Klomp J.A., Klomp J.E., Stalnecker C.A., Bryant K.L., Edwards A.C., Drizyte-Miller K., Hibshman P.S., Diehl J.N., Lee Y.S., Morales A.J. (2024). Defining the KRAS- and ERK-dependent transcriptome in KRAS-mutant cancers. Science.

[19] Luo J. (2021). KRAS mutation in pancreatic cancer. Semin. Oncol..

[20] Morris J.P., Yashinskie J.J., Koche R., Chandwani R., Tian S., Chen C.-C., Baslan T., Marinkovic Z.S., Sánchez-Rivera F.J., Leach S.D. (2019). α-Ketoglutarate links p53 to cell fate during tumour suppression. Nature.

[21] Llach J., Aguilera P., Sánchez A., Ginès A., Fernández-Esparrach G., Soy G., Sendino O., Vaquero E., Carballal S., Ausania F. (2023). Pancreatic Cancer Surveillance in Carriers of a Germline Pathogenic Variant in CDKN2A. Cancers.

[22] Dardare J., Witz A., Merlin J.-L., Gilson P., Harlé A. (2020). SMAD4 and the TGFβ Pathway in Patients with Pancreatic Ductal Adenocarcinoma. Int. J. Mol. Sci..

[23] Singhi A.D., Koay E.J., Chari S.T., Maitra A. (2019). Early Detection of Pancreatic Cancer: Opportunities and Challenges. Gastroenterology.

[24] Luo G., Jin K., Deng S., Cheng H., Fan Z., Gong Y., Qian Y., Huang Q., Ni Q., Liu C. (2021). Roles of CA19-9 in pancreatic cancer: Biomarker, predictor and promoter. Biochim. Biophys. Acta BBA Rev. Cancer.

[25] Blumenthal R.D., Leon E., Hansen H.J., Goldenberg D.M. (2007). Expression patterns of CEACAM5 and CEACAM6 in primary and metastatic cancers. BMC Cancer.

[26] Zhao B., Zhao B., Chen F. (2022). Diagnostic value of serum carbohydrate antigen 19-9 in pancreatic cancer: A systematic review and meta-analysis. Eur. J. Gastroenterol. Hepatol..

[27] Bunduc S., Gede N., Váncsa S., Lillik V., Kiss S., Juhász M.F., Erőss B., Szakács Z., Gheorghe C., Mikó A. (2022). Exosomes as prognostic biomarkers in pancreatic ductal adenocarcinoma-a systematic review and meta-analysis. Transl. Res. J. Lab. Clin. Med..

[28] Senaratne M., Swami S.S., Aye S.L., Trivedi Y., Bolgarina Z., Desai H.N., Mohammed L. (2023). Clinical Value of Circulating microRNAs in Diagnosis and Prognosis of Pancreatic Cancer: A Systematic Review. Cureus.

[29] Llop E., Guerrero P.E., Duran A., Barrabés S., Massaguer A., Ferri M.J., Albiol-Quer M., de Llorens R., Peracaula R. (2018). Glycoprotein biomarkers for the detection of pancreatic ductal adenocarcinoma. World J. Gastroenterol..

[30] Yang Y., Yan S., Tian H., Bao Y. (2018). Macrophage inhibitory cytokine-1 versus carbohydrate antigen 19-9 as a biomarker for diagnosis of pancreatic cancer: A PRISMA-compliant meta-analysis of diagnostic accuracy studies. Medicine.

[31] Kamyabi N., Bernard V., Maitra A. (2019). Liquid biopsies in pancreatic cancer. Expert Rev. Anticancer Ther..

[32] Singhi A.D., Wood L.D. (2021). Early detection of pancreatic cancer using DNA-based molecular approaches. Nat. Rev. Gastroenterol. Hepatol..

[33] Wachtel M.S., Xu K.T., Zhang Y., Chiriva-Internati M., Frezza E.E. (2008). Pancreas Cancer Survival in the Gemcitabine Era. Clin. Med. Oncol..

[34] Ciccolini J., Serdjebi C., Peters G.J., Giovannetti E. (2016). Pharmacokinetics and pharmacogenetics of Gemcitabine as a mainstay in adult and pediatric oncology: An EORTC-PAMM perspective. Cancer Chemother. Pharmacol..

[35] Skau Rasmussen L., Vittrup B., Ladekarl M., Pfeiffer P., Karen Yilmaz M., Østergaard Poulsen L., Østerlind K., Palnæs Hansen C., Bau Mortensen M., Viborg Mortensen F. (2019). The effect of postoperative gemcitabine on overall survival in patients with resected pancreatic cancer: A nationwide population-based Danish register study. Acta Oncol..

[36] Von Hoff D.D., Ervin T., Arena F.P., Chiorean E.G., Infante J., Moore M., Seay T., Tjulandin S.A., Ma W.W., Saleh M.N. (2013). Increased Survival in Pancreatic Cancer with nab-Paclitaxel plus Gemcitabine. N. Engl. J. Med..

[37] Gradishar W.J. (2006). Albumin-bound paclitaxel: A next-generation taxane. Expert Opin. Pharmacother..

[38] Kampan N.C., Madondo M.T., McNally O.M., Quinn M., Plebanski M. (2015). Paclitaxel and Its Evolving Role in the Management of Ovarian Cancer. BioMed Res. Int..

[39] Chalabi-Dchar M., Fenouil T., Machon C., Vincent A., Catez F., Marcel V., Mertani H.C., Saurin J.-C., Bouvet P., Guitton J. (2021). A novel view on an old drug, 5-fluorouracil: An unexpected RNA modifier with intriguing impact on cancer cell fate. NAR Cancer.

[40] Burris H.A., Moore M.J., Andersen J., Green M.R., Rothenberg M.L., Modiano M.R., Cripps M.C., Portenoy R.K., Storniolo A.M., Tarassoff P. (1997). Improvements in survival and clinical benefit with gemcitabine as first-line therapy for patients with advanced pancreas cancer: A randomized trial. J. Clin. Oncol. Off. J. Am. Soc. Clin. Oncol..

[41] Springfeld C., Jäger D., Büchler M.W., Strobel O., Hackert T., Palmer D.H., Neoptolemos J.P. (2019). Chemotherapy for pancreatic cancer. Presse Medicale.

[42] Conroy T., Desseigne F., Ychou M., Bouché O., Guimbaud R., Bécouarn Y., Adenis A., Raoul J.-L., Gourgou-Bourgade S., de la Fouchardière C. (2011). FOLFIRINOX versus gemcitabine for metastatic pancreatic cancer. N. Engl. J. Med..

[43] Nitipir C., Vrabie R., Parosanu A.I., Tulin R., Cretu B., Cursaru A., Slavu I., Miron A., Calu V., Orlov Slavu M.C. (2021). Clinical Impact of the Administration of FOLFIRINOX Beyond Six Months in Advanced Pancreatic Adenocarcinoma: A Cohort Study. Cureus.

[44] Carrato A., Pazo-Cid R., Macarulla T., Gallego J., Jiménez-Fonseca P., Rivera F., Cano M.T., Rodriguez-Garrote M., Pericay C., Alés I. (2024). Nab-Paclitaxel plus Gemcitabine and FOLFOX in Metastatic Pancreatic Cancer. NEJM Evid..

[45] Nichetti F., Rota S., Ambrosini P., Pircher C., Gusmaroli E., Droz Dit Busset M., Pusceddu S., Sposito C., Coppa J., Morano F. (2024). NALIRIFOX, FOLFIRINOX, and Gemcitabine with Nab-Paclitaxel as First-Line Chemotherapy for Metastatic Pancreatic Cancer: A Systematic Review and Meta-Analysis. JAMA Netw. Open.

[46] Milano G., Innocenti F., Minami H. (2022). Liposomal irinotecan (Onivyde): Exemplifying the benefits of nanotherapeutic drugs. Cancer Sci..

[47] Hudson K.L., Collins F.S. (2015). Sharing and Reporting the Results of Clinical Trials. JAMA.

[48] Rehman O.U., Fatima E., Nadeem Z.A., Azeem A., Motwani J., Imran H., Mehboob H., Khan A., Usman O. (2024). Efficacy of Cisplatin-Containing Chemotherapy Regimens in Patients of Pancreatic Ductal Adenocarcinoma: A Systematic Review and Meta-analysis. J. Gastrointest. Cancer.

[49] Crozier L., Foy R., Mouery B.L., Whitaker R.H., Corno A., Spanos C., Ly T., Gowen Cook J., Saurin A.T. (2022). CDK4/6 inhibitors induce replication stress to cause long-term cell cycle withdrawal. EMBO J..

[50] Heinrich S., Schäfer M., Weber A., Hany T.F., Bhure U., Pestalozzi B.C., Clavien P.-A. (2008). Neoadjuvant chemotherapy generates a significant tumor response in resectable pancreatic cancer without increasing morbidity: Results of a prospective phase II trial. Ann. Surg..

[51] Golcher H., Brunner T.B., Witzigmann H., Marti L., Bechstein W.-O., Bruns C., Jungnickel H., Schreiber S., Grabenbauer G.G., Meyer T. (2015). Neoadjuvant chemoradiation therapy with gemcitabine/cisplatin and surgery versus immediate surgery in resectable pancreatic cancer: Results of the first prospective randomized phase II trial. Strahlenther. Onkol..

[52] Reni M., Balzano G., Zanon S., Zerbi A., Rimassa L., Castoldi R., Pinelli D., Mosconi S., Doglioni C., Chiaravalli M. (2018). Safety and efficacy of preoperative or postoperative chemotherapy for resectable pancreatic adenocarcinoma (PACT-15): A randomised, open-label, phase 2–3 trial. Lancet Gastroenterol. Hepatol..

[53] Mackenzie M.J., Saltman D., Hirte H., Low J., Johnson C., Pond G., Moore M.J. (2007). A Phase II study of 3-aminopyridine-2-carboxaldehyde thiosemicarbazone (3-AP) and gemcitabine in advanced pancreatic carcinoma. A trial of the Princess Margaret hospital Phase II consortium. Investig. New Drugs.

[54] Attia S., Kolesar J., Mahoney M.R., Pitot H.C., Laheru D., Heun J., Huang W., Eickhoff J., Erlichman C., Holen K.D. (2008). A phase 2 consortium (P2C) trial of 3-aminopyridine-2-carboxaldehyde thiosemicarbazone (3-AP) for advanced adenocarcinoma of the pancreas. Investig. New Drugs.

[55] Martin L.K., Grecula J., Jia G., Wei L., Yang X., Otterson G.A., Wu X., Harper E., Kefauver C., Zhou B.-S. (2012). A dose escalation and pharmacodynamic study of triapine and radiation in patients with locally advanced pancreas cancer. Int. J. Radiat. Oncol. Biol. Phys..

[56] Skorupan N., Ahmad M.I., Steinberg S.M., Trepel J.B., Cridebring D., Han H., Von Hoff D.D., Alewine C. (2022). A phase II trial of the super-enhancer inhibitor Minnelide^TM^ in advanced refractory adenosquamous carcinoma of the pancreas. Future Oncol..

[57] Heumann T.R., Baretti M., Sugar E.A., Durham J.N., Linden S., Lopez-Vidal T.Y., Leatherman J., Cope L., Sharma A., Weekes C.D. (2022). A randomized, phase II trial of oral azacitidine (CC-486) in patients with resected pancreatic adenocarcinoma at high risk for recurrence. Clin. Epigenet..

[58] Kindler H.L., Hammel P., Reni M., Van Cutsem E., Macarulla T., Hall M.J., Park J.O., Hochhauser D., Arnold D., Oh D.-Y. (2022). Overall Survival Results From the POLO Trial: A Phase III Study of Active Maintenance Olaparib Versus Placebo for Germline BRCA-Mutated Metastatic Pancreatic Cancer. J. Clin. Oncol. Off. J. Am. Soc. Clin. Oncol..

[59] Golan T., Hammel P., Reni M., Van Cutsem E., Macarulla T., Hall M.J., Park J.-O., Hochhauser D., Arnold D., Oh D.-Y. (2019). Maintenance Olaparib for Germline BRCA-Mutated Metastatic Pancreatic Cancer. N. Engl. J. Med..

[60] Lian Y.-L., Lin Y.-C. (2024). The emerging tools for precisely manipulating microtubules. Curr. Opin. Cell Biol..

[61] Kaul R., Risinger A.L., Mooberry S.L. (2019). Microtubule-Targeting Drugs: More than Antimitotics. J. Nat. Prod..

[62] Gudimchuk N.B., McIntosh J.R. (2021). Regulation of microtubule dynamics, mechanics and function through the growing tip. Nat. Rev. Mol. Cell Biol..

[63] Čermák V., Dostál V., Jelínek M., Libusová L., Kovář J., Rösel D., Brábek J. (2020). Microtubule-targeting agents and their impact on cancer treatment. Eur. J. Cell Biol..

[64] Tagliamento M., Genova C., Rossi G., Coco S., Rijavec E., Dal Bello M.G., Boccardo S., Grossi F., Alama A. (2019). Microtubule-targeting agents in the treatment of non-small cell lung cancer: Insights on new combination strategies and investigational compounds. Expert Opin. Investig. Drugs.

[65] Marupudi N.I., Han J.E., Li K.W., Renard V.M., Tyler B.M., Brem H. (2007). Paclitaxel: A review of adverse toxicities and novel delivery strategies. Expert Opin. Drug Saf..

[66] Nedaeinia R., Avan A., Manian M., Salehi R., Ghayour-Mobarhan M. (2014). EGFR as a potential target for the treatment of pancreatic cancer: Dilemma and controversies. Curr. Drug Targets.

[67] Seshacharyulu P., Ponnusamy M.P., Haridas D., Jain M., Ganti A.K., Batra S.K. (2012). Targeting the EGFR signaling pathway in cancer therapy. Expert Opin. Ther. Targets.

[68] Lee C.-C., Shiao H.-Y., Wang W.-C., Hsieh H.-P. (2014). Small-molecule EGFR tyrosine kinase inhibitors for the treatment of cancer. Expert Opin. Investig. Drugs.

[69] Wang Y., Hu G., Zhang Q., Tang N., Guo J., Liu L., Han X., Wang X., Wang Z. (2016). Efficacy and safety of gemcitabine plus erlotinib for locally advanced or metastatic pancreatic cancer: A systematic review and meta-analysis. Drug Des. Devel. Ther..

[70] Liermann J., Munter M., Naumann P., Abdollahi A., Krempien R., Debus J. (2022). Cetuximab, gemcitabine and radiotherapy in locally advanced pancreatic cancer: Long-term results of the randomized controlled phase II PARC trial. Clin. Transl. Radiat. Oncol..

[71] Schultheis B., Reuter D., Ebert M.P., Siveke J., Kerkhoff A., Berdel W.E., Hofheinz R., Behringer D.M., Schmidt W.E., Goker E. (2017). Gemcitabine combined with the monoclonal antibody nimotuzumab is an active first-line regimen in KRAS wildtype patients with locally advanced or metastatic pancreatic cancer: A multicenter, randomized phase IIb study. Ann. Oncol. Off. J. Eur. Soc. Med. Oncol..

[72] Cohen M.H., Johnson J.R., Chen Y.-F., Sridhara R., Pazdur R. (2005). FDA drug approval summary: Erlotinib (Tarceva) tablets. Oncologist.

[73] Moore M.J., Goldstein D., Hamm J., Figer A., Hecht J.R., Gallinger S., Au H.J., Murawa P., Walde D., Wolff R.A. (2007). Erlotinib plus gemcitabine compared with gemcitabine alone in patients with advanced pancreatic cancer: A phase III trial of the National Cancer Institute of Canada Clinical Trials Group. J. Clin. Oncol. Off. J. Am. Soc. Clin. Oncol..

[74] Cutsem E.V., Li C.-P., Nowara E., Aprile G., Moore M., Federowicz I., Laethem J.-L.V., Hsu C., Tham C.K., Stemmer S.M. (2014). Dose escalation to rash for erlotinib plus gemcitabine for metastatic pancreatic cancer: The phase II RACHEL study. Br. J. Cancer.

[75] Crane C.H., Varadhachary G.R., Yordy J.S., Staerkel G.A., Javle M.M., Safran H., Haque W., Hobbs B.D., Krishnan S., Fleming J.B. (2011). Phase II Trial of Cetuximab, Gemcitabine, and Oxaliplatin Followed by Chemoradiation with Cetuximab for Locally Advanced (T4) Pancreatic Adenocarcinoma: Correlation of Smad4(Dpc4) Immunostaining with Pattern of Disease Progression. J. Clin. Oncol..

[76] Qin S., Li J., Bai Y., Wang Z., Chen Z., Xu R., Xu J., Zhang H., Chen J., Yuan Y. (2023). Nimotuzumab Plus Gemcitabine for K-Ras Wild-Type Locally Advanced or Metastatic Pancreatic Cancer. J. Clin. Oncol. Off. J. Am. Soc. Clin. Oncol..

[77] Shultz D.B., Pai J., Chiu W., Ng K., Hellendag M.G., Heestand G., Chang D.T., Tu D., Moore M.J., Parulekar W.R. (2016). A Novel Biomarker Panel Examining Response to Gemcitabine with or without Erlotinib for Pancreatic Cancer Therapy in NCIC Clinical Trials Group PA.3. PLoS ONE.

[78] Adamopoulos C., Cave D.D., Papavassiliou A.G. (2024). Inhibition of the RAF/MEK/ERK Signaling Cascade in Pancreatic Cancer: Recent Advances and Future Perspectives. Int. J. Mol. Sci..

[79] Frank K.J., Mulero-Sánchez A., Berninger A., Ruiz-Cañas L., Bosma A., Görgülü K., Wu N., Diakopoulos K.N., Kaya-Aksoy E., Ruess D.A. (2022). Extensive preclinical validation of combined RMC-4550 and LY3214996 supports clinical investigation for KRAS mutant pancreatic cancer. Cell Rep. Med..

[80] Ram T., Singh A.K., Kumar A., Singh H., Pathak P., Grishina M., Khalilullah H., Jaremko M., Emwas A.-H., Verma A. (2023). MEK inhibitors in cancer treatment: Structural insights, regulation, recent advances and future perspectives. RSC Med. Chem..

[81] Bodoky G., Timcheva C., Spigel D.R., La Stella P.J., Ciuleanu T.E., Pover G., Tebbutt N.C. (2012). A phase II open-label randomized study to assess the efficacy and safety of selumetinib (AZD6244 [ARRY-142886]) versus capecitabine in patients with advanced or metastatic pancreatic cancer who have failed first-line gemcitabine therapy. Investig. New Drugs.

[82] Infante J.R., Somer B.G., Park J.O., Li C.-P., Scheulen M.E., Kasubhai S.M., Oh D.-Y., Liu Y., Redhu S., Steplewski K. (2014). A randomised, double-blind, placebo-controlled trial of trametinib, an oral MEK inhibitor, in combination with gemcitabine for patients with untreated metastatic adenocarcinoma of the pancreas. Eur. J. Cancer.

[83] Hancock J.F., Magee A.I., Childs J.E., Marshall C.J. (1989). All ras proteins are polyisoprenylated but only some are palmitoylated. Cell.

[84] Kohl N.E., Wilson F.R., Mosser S.D., Giuliani E., deSolms S.J., Conner M.W., Anthony N.J., Holtz W.J., Gomez R.P., Lee T.J. (1994). Protein farnesyltransferase inhibitors block the growth of ras-dependent tumors in nude mice. Proc. Natl. Acad. Sci. USA.

[85] Gibbs J.B., Oliff A., Kohl N.E. (1994). Farnesyltransferase inhibitors: Ras research yields a potential cancer therapeutic. Cell.

[86] Van Cutsem E., van de Velde H., Karasek P., Oettle H., Vervenne W.L., Szawlowski A., Schoffski P., Post S., Verslype C., Neumann H. (2004). Phase III Trial of Gemcitabine Plus Tipifarnib Compared with Gemcitabine Plus Placebo in Advanced Pancreatic Cancer. J. Clin. Oncol..

[87] Baranyi M., Buday L., Hegedűs B. (2020). K-Ras prenylation as a potential anticancer target. Cancer Metastasis Rev..

[88] Rudloff U. (2022). Emerging kinase inhibitors for the treatment of pancreatic ductal adenocarcinoma. Expert Opin. Emerg. Drugs.

[89] Yang Y., Li S., Wang Y., Zhao Y., Li Q. (2022). Protein tyrosine kinase inhibitor resistance in malignant tumors: Molecular mechanisms and future perspective. Signal Transduct. Target. Ther..

[90] Kumar V., Kaur N., Sahu S., Sharma V., Kumar D., Sharma A., Wadhwa P. (2023). Role of Tyrosine Kinases and their Inhibitors in Cancer Therapy: A Comprehensive Review. Curr. Med. Chem..

[91] Deplanque G., Demarchi M., Hebbar M., Flynn P., Melichar B., Atkins J., Nowara E., Moyé L., Piquemal D., Ritter D. (2015). A randomized, placebo-controlled phase III trial of masitinib plus gemcitabine in the treatment of advanced pancreatic cancer. Ann. Oncol. Off. J. Eur. Soc. Med. Oncol..

[92] Gunderson A.J., Kaneda M.M., Tsujikawa T., Nguyen A.V., Affara N.I., Ruffell B., Gorjestani S., Liudahl S.M., Truitt M., Olson P. (2016). Bruton Tyrosine Kinase-Dependent Immune Cell Cross-talk Drives Pancreas Cancer. Cancer Discov..

[93] Sinha M., Betts C., Zhang L., Griffith M.J., Solman I., Chen B., Liu E., Tamaki W., Stultz J., Marquez J. (2023). Modulation of myeloid and T cells in vivo by Bruton’s tyrosine kinase inhibitor ibrutinib in patients with metastatic pancreatic ductal adenocarcinoma. J. Immunother. Cancer.

[94] Tempero M., Oh D.-Y., Tabernero J., Reni M., Van Cutsem E., Hendifar A., Waldschmidt D.-T., Starling N., Bachet J.-B., Chang H.-M. (2021). Ibrutinib in combination with nab-paclitaxel and gemcitabine for first-line treatment of patients with metastatic pancreatic adenocarcinoma: Phase III RESOLVE study. Ann. Oncol. Off. J. Eur. Soc. Med. Oncol..

[95] Cortes J.E., Saglio G., Kantarjian H.M., Baccarani M., Mayer J., Boqué C., Shah N.P., Chuah C., Casanova L., Bradley-Garelik B. (2016). Final 5-Year Study Results of DASISION: The Dasatinib Versus Imatinib Study in Treatment-Naïve Chronic Myeloid Leukemia Patients Trial. J. Clin. Oncol. Off. J. Am. Soc. Clin. Oncol..

[96] Chee C.E., Krishnamurthi S., Nock C.J., Meropol N.J., Gibbons J., Fu P., Bokar J., Teston L., O’Brien T., Gudena V. (2013). Phase II Study of Dasatinib (BMS-354825) in Patients with Metastatic Adenocarcinoma of the Pancreas. Oncologist.

[97] George T.J., Starr J.S., Parekh H.D., Ivey A.M., McGorray S.P., Wang Y., Dang L.H., Daily K.C., Allegra C.J., DeRemer D.L. (2018). Final results from a phase II study of 5-fluorouracil, oxaliplatin, and dasatinib (FOLFOX-D) in previously untreated metastatic pancreatic adenocarcinoma. J. Clin. Oncol..

[98] Evans T.R.J., Cutsem E.V., Moore M.J., Bazin I.S., Rosemurgy A., Bodoky G., Deplanque G., Harrison M., Melichar B., Pezet D. (2017). Phase 2 placebo-controlled, double-blind trial of dasatinib added to gemcitabine for patients with locally-advanced pancreatic cancer. Ann. Oncol..

[99] Lu Z., Weniger M., Jiang K., Boeck S., Zhang K., Bazhin A., Miao Y., Werner J., D’Haese J.G. (2018). Therapies Targeting the Tumor Stroma and the VEGF/VEGFR Axis in Pancreatic Ductal Adenocarcinoma: A Systematic Review and Meta-Analysis. Target. Oncol..

[100] Kindler H.L., Ioka T., Richel D.J., Bennouna J., Létourneau R., Okusaka T., Funakoshi A., Furuse J., Park Y.S., Ohkawa S. (2011). Axitinib plus gemcitabine versus placebo plus gemcitabine in patients with advanced pancreatic adenocarcinoma: A double-blind randomised phase 3 study. Lancet Oncol..

[101] Wu H., Fu M., Wu M., Cao Z., Zhang Q., Liu Z. (2024). Emerging mechanisms and promising approaches in pancreatic cancer metabolism. Cell Death Dis..

[102] Mossmann D., Park S., Hall M.N. (2018). mTOR signalling and cellular metabolism are mutual determinants in cancer. Nat. Rev. Cancer.

[103] Ferro F., Servais S., Besson P., Roger S., Dumas J.-F., Brisson L. (2020). Autophagy and mitophagy in cancer metabolic remodelling. Semin. Cell Dev. Biol..

[104] Philip P.A., Buyse M.E., Alistar A.T., Rocha Lima C.M., Luther S., Pardee T.S., Van Cutsem E. (2019). A Phase III open-label trial to evaluate efficacy and safety of CPI-613 plus modified FOLFIRINOX (mFFX) versus FOLFIRINOX (FFX) in patients with metastatic adenocarcinoma of the pancreas. Future Oncol..

[105] Wolpin B.M., Hezel A.F., Abrams T., Blaszkowsky L.S., Meyerhardt J.A., Chan J.A., Enzinger P.C., Allen B., Clark J.W., Ryan D.P. (2009). Oral mTOR Inhibitor Everolimus in Patients with Gemcitabine-Refractory Metastatic Pancreatic Cancer. J. Clin. Oncol..

[106] Chung V., McDonough S., Philip P.A., Cardin D., Wang-Gillam A., Hui L., Tejani M.A., Seery T.E., Dy I.A., Al Baghdadi T. (2017). Effect of Selumetinib and MK-2206 vs Oxaliplatin and Fluorouracil in Patients with Metastatic Pancreatic Cancer after Prior Therapy: SWOG S1115 Study Randomized Clinical Trial. JAMA Oncol..

[107] Debnath J., Gammoh N., Ryan K.M. (2023). Autophagy and autophagy-related pathways in cancer. Nat. Rev. Mol. Cell Biol..

[108] Assi M., Kimmelman A.C. (2023). Impact of context-dependent autophagy states on tumor progression. Nat. Cancer.

[109] Vempati R.K., Malla R.R. (2020). Autophagy-Induced Drug Resistance in Liver Cancer. Crit. Rev. Oncog..

[110] Fei N., Wen S., Ramanathan R., Hogg M.E., Zureikat A.H., Lotze M.T., Bahary N., Singhi A.D., Zeh H.J., Boone B.A. (2021). SMAD4 loss is associated with response to neoadjuvant chemotherapy plus hydroxychloroquine in patients with pancreatic adenocarcinoma. Clin. Transl. Sci..

[111] De Santis M.C., Bockorny B., Hirsch E., Cappello P., Martini M. (2024). Exploiting pancreatic cancer metabolism: Challenges and opportunities. Trends Mol. Med..

[112] Fontes M.G., Silva C., Roldán W.H., Monteiro G. (2024). Exploring the potential of asparagine restriction in solid cancer treatment: Recent discoveries, therapeutic implications, and challenges. Med. Oncol..

[113] Burke M.J., Zalewska-Szewczyk B. (2022). Hypersensitivity reactions to asparaginase therapy in acute lymphoblastic leukemia: Immunology and clinical consequences. Future Oncol..

[114] Hammel P., Fabienne P., Mineur L., Metges J.-P., Andre T., De La Fouchardiere C., Louvet C., El Hajbi F., Faroux R., Guimbaud R. (2020). Erythrocyte-encapsulated asparaginase (eryaspase) combined with chemotherapy in second-line treatment of advanced pancreatic cancer: An open-label, randomized Phase IIb trial. Eur. J. Cancer.

[115] Hammel P., El-Hariry I., Macarulla T., Garcia-Carbonero R., Metges J.-P., Bouché O., Portales F., Pazo Cid R.A., Mineur L., Cubillo Gracian A.M. (2022). Trybeca-1: A randomized, phase 3 study of eryaspase in combination with chemotherapy versus chemotherapy alone as second-line treatment in patients with advanced pancreatic adenocarcinoma (NCT03665441). J. Clin. Oncol..

[116] Nishikawa H., Goto M., Fukunishi S., Asai A., Nishiguchi S., Higuchi K. (2021). Cancer Cachexia: Its Mechanism and Clinical Significance. Int. J. Mol. Sci..

[117] Neshan M., Tsilimigras D.I., Han X., Zhu H., Pawlik T.M. (2024). Molecular Mechanisms of Cachexia: A Review. Cells.

[118] Yu S.-Y., Luan Y., Dong R., Abazarikia A., Kim S.-Y. (2022). Adipose Tissue Wasting as a Determinant of Pancreatic Cancer-Related Cachexia. Cancers.

[119] Robinson T.P., Hamidi T., Counts B., Guttridge D.C., Ostrowski M.C., Zimmers T.A., Koniaris L.G. (2023). The impact of inflammation and acute phase activation in cancer cachexia. Front. Immunol..

[120] Bowers M., Cucchiaro B., Reid J., Slee A. (2023). Non-steroidal anti-inflammatory drugs for treatment of cancer cachexia: A systematic review. J. Cachexia Sarcopenia Muscle.

[121] Paval D.R., Patton R., McDonald J., Skipworth R.J.E., Gallagher I.J., Laird B.J. (2022). A systematic review examining the relationship between cytokines and cachexia in incurable cancer. J. Cachexia Sarcopenia Muscle.

[122] Golan T., Geva R., Richards D., Madhusudan S., Lin B.K., Wang H.T., Walgren R.A., Stemmer S.M. (2018). LY2495655, an antimyostatin antibody, in pancreatic cancer: A randomized, phase 2 trial. J. Cachexia Sarcopenia Muscle.

[123] McCroskery S., Thomas M., Maxwell L., Sharma M., Kambadur R. (2003). Myostatin negatively regulates satellite cell activation and self-renewal. J. Cell Biol..

[124] Borad M.J., Reddy S.G., Bahary N., Uronis H.E., Sigal D., Cohn A.L., Schelman W.R., Stephenson J., Chiorean E.G., Rosen P.J. (2015). Randomized Phase II Trial of Gemcitabine Plus TH-302 Versus Gemcitabine in Patients with Advanced Pancreatic Cancer. J. Clin. Oncol. Off. J. Am. Soc. Clin. Oncol..

[125] Van Cutsem E., Lenz H.-J., Furuse J., Tabernero J., Heinemann V., Ioka T., Bazin I., Ueno M., Csõszi T., Wasan H. (2016). MAESTRO: A randomized, double-blind phase III study of evofosfamide (Evo) in combination with gemcitabine (Gem) in previously untreated patients (pts) with metastatic or locally advanced unresectable pancreatic ductal adenocarcinoma (PDAC). J. Clin. Oncol..

[126] Lu C., Talukder A., Savage N.M., Singh N., Liu K. (2017). JAK-STAT-mediated chronic inflammation impairs cytotoxic T lymphocyte activation to decrease anti-PD-1 immunotherapy efficacy in pancreatic cancer. OncoImmunology.

[127] Zitvogel L., Galluzzi L., Kepp O., Smyth M.J., Kroemer G. (2015). Type I interferons in anticancer immunity. Nat. Rev. Immunol..

[128] Ruan Q., Wen C., Jin G., Yuan Z., Yang X., Wen Z., Huang G., Li G., Deng J., Bai Y. (2023). Phloretin-induced STAT3 inhibition suppresses pancreatic cancer growth and progression via enhancing Nrf2 activity. Phytomed. Int. J. Phytother. Phytopharm..

[129] Hurwitz H.I., Uppal N., Wagner S.A., Bendell J.C., Beck J.T., Wade S.M., Nemunaitis J.J., Stella P.J., Pipas J.M., Wainberg Z.A. (2015). Randomized, Double-Blind, Phase II Study of Ruxolitinib or Placebo in Combination with Capecitabine in Patients with Metastatic Pancreatic Cancer for Whom Therapy with Gemcitabine Has Failed. J. Clin. Oncol. Off. J. Am. Soc. Clin. Oncol..

[130] Bekaii-Saab T., Okusaka T., Goldstein D., Oh D.-Y., Ueno M., Ioka T., Fang W., Anderson E.C., Noel M.S., Reni M. (2023). Napabucasin plus nab-paclitaxel with gemcitabine versus nab-paclitaxel with gemcitabine in previously untreated metastatic pancreatic adenocarcinoma: An adaptive multicentre, randomised, open-label, phase 3, superiority trial. EClinicalMedicine.

[131] Chen J., Wang J., Xu H. (2021). Comparison of atezolizumab, durvalumab, pembrolizumab, and nivolumab as first-line treatment in patients with extensive-stage small cell lung cancer: A systematic review and network meta-analysis. Medicine.

[132] Pei R., Shi Y., Lv S., Dai T., Zhang F., Liu S., Wu B. (2021). Nivolumab vs Pembrolizumab for Treatment of US Patients with Platinum-Refractory Recurrent or Metastatic Head and Neck Squamous Cell Carcinoma: A Network Meta-analysis and Cost-effectiveness Analysis. JAMA Netw. Open.

[133] Malmberg R., Zietse M., Dumoulin D.W., Hendrikx J.J.M.A., Aerts J.G.J.V., van der Veldt A.A.M., Koch B.C.P., Sleijfer S., van Leeuwen R.W.F. (2022). Alternative dosing strategies for immune checkpoint inhibitors to improve cost-effectiveness: A special focus on nivolumab and pembrolizumab. Lancet Oncol..

[134] Sarfraz Z., Sarfraz A., Farooq M.D., Khalid M., Cheema K., Javad F., Khan T., Pervaiz Z., Sarfraz M., Jaan A. (2024). The Current Landscape of Clinical Trials for Immunotherapy in Pancreatic Cancer: A State-of-the-Art Review. J. Gastrointest. Cancer.

[135] Singh G., Kutcher D., Lally R., Rai V. (2024). Targeting Neoantigens in Pancreatic Ductal Adenocarcinoma. Cancers.

[136] Farhangnia P., Khorramdelazad H., Nickho H., Delbandi A.-A. (2024). Current and future immunotherapeutic approaches in pancreatic cancer treatment. J. Hematol. Oncol..

[137] Do C.T., Prochnau J.Y., Dominguez A., Wang P., Rao M.K. The Road Ahead in Pancreatic Cancer: Emerging Trends and Therapeutic Prospects Biomedicines 2024, 12, 1979. 12.

[138] Bockorny B., Semenisty V., Macarulla T., Borazanci E., Wolpin B.M., Stemmer S.M., Golan T., Geva R., Borad M.J., Pedersen K.S. (2020). BL-8040, a CXCR4 antagonist, in combination with pembrolizumab and chemotherapy for pancreatic cancer: The COMBAT trial. Nat. Med..

[139] Canel M., Sławińska A.D., Lonergan D.W., Kallor A.A., Upstill-Goddard R., Davidson C., von Kriegsheim A., Biankin A.V., Byron A., Alfaro J. (2023). FAK suppresses antigen processing and presentation to promote immune evasion in pancreatic cancer. Gut.

[140] Lander V.E., Belle J.I., Kingston N.L., Herndon J.M., Hogg G.D., Liu X., Kang L.-I., Knolhoff B.L., Bogner S.J., Baer J.M. (2022). Stromal Reprogramming by FAK Inhibition Overcomes Radiation Resistance to Allow for Immune Priming and Response to Checkpoint Blockade. Cancer Discov..

[141] Gong N., Alameh M.-G., El-Mayta R., Xue L., Weissman D., Mitchell M.J. (2024). Enhancing in situ cancer vaccines using delivery technologies. Nat. Rev. Drug Discov..

[142] Guasp P., Reiche C., Sethna Z., Balachandran V.P. (2024). RNA vaccines for cancer: Principles to practice. Cancer Cell.

[143] Tsujikawa T., Crocenzi T., Durham J.N., Sugar E.A., Wu A.A., Onners B., Nauroth J.M., Anders R.A., Fertig E.J., Laheru D.A. (2020). Evaluation of Cyclophosphamide/GVAX Pancreas Followed by Listeria-Mesothelin (CRS-207) with or without Nivolumab in Patients with Pancreatic Cancer. Clin. Cancer Res. Off. J. Am. Assoc. Cancer Res..

[144] Le D.T., Wang-Gillam A., Picozzi V., Greten T.F., Crocenzi T., Springett G., Morse M., Zeh H., Cohen D., Fine R.L. (2015). Safety and survival with GVAX pancreas prime and Listeria Monocytogenes-expressing mesothelin (CRS-207) boost vaccines for metastatic pancreatic cancer. J. Clin. Oncol. Off. J. Am. Soc. Clin. Oncol..

[145] Parikh K., Banna G., Liu S.V., Friedlaender A., Desai A., Subbiah V., Addeo A. (2022). Drugging KRAS: Current perspectives and state-of-art review. J. Hematol. Oncol. J. Hematol. Oncol..

[146] Hong D.S., Fakih M.G., Strickler J.H., Desai J., Durm G.A., Shapiro G.I., Falchook G.S., Price T.J., Sacher A., Denlinger C.S. (2020). KRASG12C Inhibition with Sotorasib in Advanced Solid Tumors. N. Engl. J. Med..

[147] Punekar S.R., Velcheti V., Neel B.G., Wong K.-K. (2022). The current state of the art and future trends in RAS-targeted cancer therapies. Nat. Rev. Clin. Oncol..

[148] Ostrem J.M., Peters U., Sos M.L., Wells J.A., Shokat K.M. (2013). K-Ras(G12C) inhibitors allosterically control GTP affinity and effector interactions. Nature.

[149] Skoulidis F., Li B.T., Dy G.K., Price T.J., Falchook G.S., Wolf J., Italiano A., Schuler M., Borghaei H., Barlesi F. (2021). Sotorasib for Lung Cancers with KRAS p.G12C Mutation. N. Engl. J. Med..

[150] Jänne P.A., Riely G.J., Gadgeel S.M., Heist R.S., Ou S.-H.I., Pacheco J.M., Johnson M.L., Sabari J.K., Leventakos K., Yau E. (2022). Adagrasib in Non-Small-Cell Lung Cancer Harboring a KRASG12C Mutation. N. Engl. J. Med..

[151] Zhou C., Li C., Luo L., Li X., Jia K., He N., Mao S., Wang W., Shao C., Liu X. (2024). Anti-tumor efficacy of HRS-4642 and its potential combination with proteasome inhibition in KRAS G12D-mutant cancer. Cancer Cell.

[152] Hallin J., Bowcut V., Calinisan A., Briere D.M., Hargis L., Engstrom L.D., Laguer J., Medwid J., Vanderpool D., Lifset E. (2022). Anti-tumor efficacy of a potent and selective non-covalent KRASG12D inhibitor. Nat. Med..

[153] Jiang J., Jiang L., Maldonato B.J., Wang Y., Holderfield M., Aronchik I., Winters I.P., Salman Z., Blaj C., Menard M. (2024). Translational and Therapeutic Evaluation of RAS-GTP Inhibition by RMC-6236 in RAS-Driven Cancers. Cancer Discov..

[154] Holderfield M., Lee B.J., Jiang J., Tomlinson A., Seamon K.J., Mira A., Patrucco E., Goodhart G., Dilly J., Gindin Y. (2024). Concurrent inhibition of oncogenic and wild-type RAS-GTP for cancer therapy. Nature.

[155] Wasko U.N., Jiang J., Dalton T.C., Curiel-Garcia A., Edwards A.C., Wang Y., Lee B., Orlen M., Tian S., Stalnecker C.A. (2024). Tumour-selective activity of RAS-GTP inhibition in pancreatic cancer. Nature.

[156] Piersma B., Hayward M.-K., Weaver V.M. (2020). Fibrosis and cancer: A strained relationship. Biochim. Biophys. Acta Rev. Cancer.

[157] Jiang H., Hegde S., Knolhoff B.L., Zhu Y., Herndon J.M., Meyer M.A., Nywening T.M., Hawkins W.G., Shapiro I.M., Weaver D.T. (2016). Targeting focal adhesion kinase renders pancreatic cancers responsive to checkpoint immunotherapy. Nat. Med..

[158] Serpas V.J., Raghav K.P., Halperin D.M., Yao J., Overman M.J. (2018). Discrepancies in endpoints between clinical trial protocols and clinical trial registration in randomized trials in oncology. BMC Med. Res. Methodol..

[159] Shamliyan T.A., Kane R.L. (2014). Availability of results from clinical research: Failing policy efforts. J. Epidemiol. Glob. Health.

[160] Gill C.J. (2012). How often do US-based human subjects research studies register on time, and how often do they post their results? A statistical analysis of the Clinicaltrials.gov database. BMJ Open.

[161] Tse T., Fain K.M., Zarin D.A. (2018). How to avoid common problems when using ClinicalTrials.gov in research: 10 issues to consider. BMJ.

[162] Miller J.E., Wilenzick M., Ritcey N., Ross J.S., Mello M.M. (2017). Measuring clinical trial transparency: An empirical analysis of newly approved drugs and large pharmaceutical companies. BMJ Open.

[163] Mayo-Wilson E., Heyward J., Keyes A., Reynolds J., White S., Atri N., Alexander C., Omar A., Ford D.E. (2018). Clinical trial registration and reporting: A survey of academic organizations in the United States. BMC Med..

[164] Daamen L.A., Molenaar I.Q., Groot V.P. (2023). Recent Advances and Future Challenges in Pancreatic Cancer Care: Early Detection, Liquid Biopsies, Precision Medicine and Artificial Intelligence. J. Clin. Med..

[165] Mukund A., Afridi M.A., Karolak A., Park M.A., Permuth J.B., Rasool G. (2024). Pancreatic Ductal Adenocarcinoma (PDAC): A Review of Recent Advancements Enabled by Artificial Intelligence. Cancers.

[166] Cao K., Xia Y., Yao J., Han X., Lambert L., Zhang T., Tang W., Jin G., Jiang H., Fang X. (2023). Large-scale pancreatic cancer detection via non-contrast CT and deep learning. Nat. Med..

[167] Barbey O., Lulka H., Hanoun N., Belhadj-Tahar H., Vernejoul F., Cambois G., Tiraby M., Buscail L., Gross F., Cordelier P. (2023). Preclinical development of non-viral gene therapy for patients with advanced pancreatic cancer. Mol. Ther. Methods Clin. Dev..

[168] Hosein A.N., Brekken R.A., Maitra A. (2020). Pancreatic cancer stroma: An update on therapeutic targeting strategies. Nat. Rev. Gastroenterol. Hepatol..

[169] Cox A.D., Der C.J. (2024). KRAS takes the road to destruction. Science.

[170] Bannoura S.F., Uddin M.H., Nagasaka M., Fazili F., Al-Hallak M.N., Philip P.A., El-Rayes B., Azmi A.S. (2021). Targeting KRAS in pancreatic cancer: New drugs on the horizon. Cancer Metastasis Rev..

[171] Kumarasamy V., Wang J., Frangou C., Wan Y., Dynka A., Rosenheck H., Dey P., Abel E.V., Knudsen E.S., Witkiewicz A.K. (2024). The Extracellular Niche and Tumor Microenvironment Enhance KRAS Inhibitor Efficacy in Pancreatic Cancer. Cancer Res..

[172] Kemp S.B., Cheng N., Markosyan N., Sor R., Kim I.-K., Hallin J., Shoush J., Quinones L., Brown N.V., Bassett J.B. (2023). Efficacy of a Small-Molecule Inhibitor of KrasG12D in Immunocompetent Models of Pancreatic Cancer. Cancer Discov..

[173] Kim D., Herdeis L., Rudolph D., Zhao Y., Böttcher J., Vides A., Ayala-Santos C.I., Pourfarjam Y., Cuevas-Navarro A., Xue J.Y. (2023). Pan-KRAS inhibitor disables oncogenic signalling and tumour growth. Nature.

[174] Popow J., Farnaby W., Gollner A., Kofink C., Fischer G., Wurm M., Zollman D., Wijaya A., Mischerikow N., Hasenoehrl C. (2024). Targeting cancer with small-molecule pan-KRAS degraders. Science.

